# Systematic Review and Meta-Analysis of Current and Novel Approaches in the Management of Borderline Resectable and Locally Advanced Pancreatic Cancer

**DOI:** 10.3390/cancers18071139

**Published:** 2026-04-01

**Authors:** Kelvin Le, Khang Duy Ricky Le, Wei Hong, Peter Gibbs, Osamu Yoshino, Belinda Lee

**Affiliations:** 1 Faculty of Medicine, Dentistry and Health Sciences, Melbourne Medical School, University of Melbourne, Parkville, VIC 3010, Australia; 2 Walter & Eliza Hall Institute (WEHI), Parkville, VIC 3050, Australia; 3 Royal Melbourne Hospital, Parkville, VIC 3050, Australia; 4 School of Medicine, Faculty of Health, Deakin University, Geelong, VIC 3220, Australia; 5 St Vincent’s Hospital, Fitzroy, VIC 3065, Australia; 6 Austin Hospital, Heidelberg, VIC 3084, Australia; 7 Peter MacCallum Cancer Centre, Melbourne, VIC 3000, Australia; 8 Northern Hospital, Epping, VIC 3076, Australia

**Keywords:** borderline resectable, locally advanced, pancreatic ductal adenocarcinoma, neoadjuvant, resection, downstage, survival, recurrence, chemotherapy, chemoradiotherapy

## Abstract

**Background**: Pancreatic ductal adenocarcinoma (PDAC) is a leading cause of cancer-related mortality with poor survival outcomes. Resection is the sole definitive management; however, most PDACs are diagnosed with unresectable disease including metastatic, locally advanced (LAPC) or borderline resectable (BRPC). Recently, neoadjuvant therapy has demonstrated potential in downstaging these tumours for resection. This literature review explores current and novel approaches in the management of BRPC and LAPC. **Methods**: A systematic search of Medline, Embase, Cochrane Central and Emcare databases was conducted on 7 April 2025. Inclusion criteria were primary articles that explored current and novel therapies that led to downstaging of BRPC and LAPC to resection, as well as resection outcomes and oncological outcomes associated with this. Articles that explored other pancreatic cancer subtypes or either resectable or metastatic disease were excluded. All meta-analyses were performed using a random-effects model based on the inverse variance method. **Results**: A total of 88 studies involving 8585 patients were included in the review, predominately from retrospective studies (57%, *n* = 50). Neoadjuvant regimens incorporating chemotherapy or radiotherapy, whether sequential or concurrent, demonstrated the highest proportions of R0 resections with N0 status. Overall, most modalities showed evidence of survival benefit following resection compared to non-operative management, with pooled differences demonstrated for chemotherapy alone (HR 0.33, 95% CI 0.25–0.44) and sequential chemotherapy and radiotherapy (HR 0.49, 95% CI 0.25–0.95). However, no significant differences between these modalities were demonstrated. Other modality-specific conclusions regarding survival benefit could not be elucidated. **Conclusions**: The rising incidence and global mortality from PDAC underscore the significance of identifying approaches to optimise the management of BRPC and LAPC. This review emphasises the importance of neoadjuvant therapy, both current and novel with surgical resection, which may warrant further investigation in future clinical trials. However, it is important to acknowledge the clinical heterogeneity of current data, which may introduce bias. Nevertheless, these findings can help to inform guidelines on the management of BRPC and LAPC.

## Simple Summary

Resection remains fundamental to the curative-intent management of pancreatic cancer. However, opportunities for resection are limited in borderline resectable (BRPC) and locally advanced (LAPC) pancreatic cancer due to tumour involvement of the surrounding vasculature. Neoadjuvant therapy shows promise in improving survival, aiming to potentially ‘downstage’ BRPC and LAPC by reducing tumour size, vascular involvement and improving systemic control of micrometastatic disease. This could involve multimodal chemotherapy with or without radiotherapy, as well as newer modalities including irreversible electroporation, targeted therapy and immunotherapy. This systematic review and meta-analysis of 88 articles demonstrated improved surgical outcomes following regimens that incorporated chemotherapy and radiotherapy, whether concurrent or sequential. Resection following chemotherapy alone or sequential chemotherapy and radiotherapy improved overall survival compared to non-operative management; however, there were no differences in effect between these modalities. Other modality-specific impacts on survival could not be elucidated. Future studies are warranted to improve BRPC and LAPC outcomes.

## 1. Introduction

Pancreatic cancer, most commonly pancreatic ductal adenocarcinoma (PDAC), is an aggressive cancer with one of the worst survival outcomes. It is the sixth leading cause of cancer-related mortality worldwide, with a poor 5-year survival rate of up to 13% [1,2,3]. Surgical resection is a central component of multimodality treatment for localised PDAC with proven significant survival benefits if effectively and holistically delivered [4]. However, opportunities for resection are limited in more advanced localised PDAC, namely borderline resectable (BRPC) and locally advanced (LAPC) pancreatic cancer, which comprise up to 40% of initial diagnoses [5,6,7].

The management of BRPC and LAPC is complicated by staging and operative challenges. Currently, there is no consensus on the diagnostic staging criteria of BRPC and LAPC. Conventional staging of resectability is radiologically determined by the extent of tumour encroachment to surrounding vasculature including the coeliac trunk, common hepatic artery (CHA), superior mesenteric artery (SMA), superior mesenteric vein (SMV) and portal vein (PV) without distant metastasis [7,8]. Guidelines defining resectability have been recommended by various institutions and cooperative bodies [9,10,11,12,13,14,15,16]. Subtle differences in these staging definitions may influence subsequent management and treatment outcomes [17]. Beyond the variability in diagnostic guidelines, staging and management are also challenged by systemic tumour involvement that is undetectable by conventional cross-sectional imaging [4]. A recent study demonstrated that the incidence of intraabdominal occult metastasis in all non-metastatic PDAC was 8%, of which BRPC or LAPC after neoadjuvant therapy was 31% after age and gender matching [18]. More specific to BRPC and LAPC, treatment is complicated due to the large tumour size and extensive vascular involvement with potentially undetectable systemic disease. More radical resection involving additional vascular resection and reconstruction are required compared to classic pancreaticoduodenectomy to achieve a margin-negative status [19,20]. Yet, high rates of margin-positive resections are still reported due to the infiltrative nature of advanced localised PDAC and their difficulty detecting microscopic margins, which increases the susceptibility to local or systemic recurrence [8]. Additionally, these complex procedures are associated with higher rates of morbidity and mortality [21].

Despite these challenges, neoadjuvant approaches show potential in reducing tumour size, vascular involvement and systemic involvement of more advanced localised PDAC for surgical reassessment. This is often defined as downstaging to grant patients opportunities for margin-negative resection with the goal to improve oncological outcomes [4,22,23,24]. Previous systematic reviews report resection rates following neoadjuvant therapies up to 67.8% for BRPC candidates and 28% for LAPC candidates, with margin-negative resection rates of up to 83.9% and 77% respectively [25,26,27]. Resection following multimodal neoadjuvant therapy has demonstrated improvement in survival outcomes for those that complete curative-intent surgery [23,28]. However, a large proportion of patients with BRPC and especially LAPC do not demonstrate adequate downstaging responses to neoadjuvant therapies to warrant the decision to resect [25,26,27]. Furthermore, there is no consensus for the most optimal approach for patient selection for neoadjuvant therapy, neoadjuvant regimen and its length, treatment modality or timing of surgery during neoadjuvant treatment [29]. Determining the optimal neoadjuvant modalities that will allow patients the best chance of downstaging for resection is crucial to improving outcomes in PDAC. Current approaches to downstaging BRPC and LAPC involve neoadjuvant combination chemotherapy regimens such as FOLFIRINOX (fluorouracil, leucovorin, irinotecan and oxaliplatin) or gemcitabine-based therapies with or without chemoradiotherapy [4,5,22]. Numerous permutations with multimodal neoadjuvant approaches for managing BRPC and LAPC have been conducted in the past decade, including various regimens of chemotherapy, sequential or concurrent chemoradiotherapy with external-beam radiation therapy (EBRT), stereotactic body radiation therapy (SBRT) and/or intensity-modulated radiation therapy (IMRT). More recently, clinical trials of novel approaches including irreversible electroporation (IRE), targeted therapy and immunotherapy have emerged [4,5,8,30,31,32]. Together, the emergence of novel neoadjuvant approaches offers new potential for improving BRPC and LAPC survival outcomes.

This systematic review provides an up-to-date evidence-based overview of current and novel neoadjuvant approaches in the management of BRPC and LAPC to improve survival.

## 2. Materials and Methods

### 2.1. Literature Search Strategy

This systematic review was performed in accordance with the Preferred Reporting Items for Systematic Reviews and Meta-Analyses (PRISMA) guidelines and has not been registered [33] (Supplementary Table S20). A computer-assisted literature search was performed on Medline, Embase, Cochrane Central and Emcare databases on 7 April 2025. Additional studies were screened from reference lists of relevant articles for further inclusion in this review. The search strategy combined Boolean operators and medical subject headings (MeSH) terms related to BRPC or LAPC and outcomes following treatment. The complete search strategy is provided in the Appendix A.

### 2.2. Eligibility Criteria

Peer-reviewed full-text articles available in the English language that evaluated outcomes related to current management of BRPC and LAPC with neoadjuvant therapy and resection were considered in this review.

Articles were included if they were of the following study designs: original randomised controlled trials, retrospective or prospective cohort studies, retrospective or prospective case–control studies and retrospective or prospective observational studies. Additional inclusion criteria were articles that evaluated adult populations (age > 18 years) with PDAC, articles published within the past 10 years, and relating to neoadjuvant management of BRPC/LAPC including chemotherapy, radiotherapy, chemoradiotherapy, IRE, targeted therapy, immunotherapy or a combination of these modalities followed by surgery.

Articles were excluded if they were of the following study designs: mixed-method studies, abstracts, conference papers, systematic and non-systematic reviews or meta-analyses, commentaries, letters, editorials, opinion articles, study protocols, case reports and non-human trials. Additional exclusion criteria included articles that evaluated pancreatic cancer of other subtypes such as squamous carcinomas and neuroendocrine tumours, as well as metastatic or resectable pancreatic cancers.

### 2.3. Literature Screening

Screening of articles was performed using the Covidence systematic review management application. Initial screening by title and abstract was performed by two independent investigators (KL, KDRL). Articles that met inclusion criteria or had insufficient information to determine their eligibility progressed to full-text analysis. This was subsequently performed independently by the same investigators. Disagreement during this process was resolved by discussion and consensus (KL, KDRL, OY, BL).

### 2.4. Endpoints

The outcomes of this review were related to understanding the current landscape of management for BRPC and LAPC.

The primary outcome therefore include the following:

1. Outcomes of downstaging to surgical resection following neoadjuvant therapy, including objective response rate (ORR; including partial response PR or complete response CR), rates of proceeding to surgery and rates of resection.
2. Resection outcomes following neoadjuvant therapy, including resection status (R0 denoting margin-negative resection) and nodal status (degree of nodal metastasis; N0 denoting no nodal involvement).

The secondary outcomes were as follows:

1. Oncological outcomes, including overall survival (OS), recurrence-free survival (RFS) and progression-free survival (PFS) rates for patients who did or did not undergo surgical intervention.

### 2.5. Data Extraction

Included articles were extracted (KL, KDRL) for fundamental identifiers including author, year and country of publication, study design and sample size. Additional demographic identifiers such as age and sex of participants, treatment modality and outcomes of interest were extracted.

### 2.6. Risk-of-Bias and Quality Assessment

Risk-of-bias and quality assessments of included studies were performed using Version 2 of the Cochrane Risk-of-Bias Tool for Randomised Trials (RoB 2) [34] for randomised controlled trials, the Methodological Index for Non-Randomised Studies (MINORS) [35] for single-arm, non-comparative studies and the Risk of Bias in Non-Randomised Studies of Interventions (ROBINS-I) tool [36] for non-randomised studies by two independent investigators (KL, KDRL). For the MINORS tool, scores < 9, between 9 and 12, and >12 were considered low, moderate and high quality, respectively. Disagreement during this process was resolved by discussion and consensus. Risk-of-bias visualisations were performed using the robvis tool [37]. The certainty-of-evidence assessment was performed using the Grading of Recommendations Assessment, Development and Evaluation (GRADE) framework [38] by two independent investigators (KL, KDRL). Disagreement during this process was resolved by discussion and consensus.

### 2.7. Data Synthesis and Statistical Analysis

Outcome data were synthesised systematically according to the endpoints of interest. Statistical analyses were performing using R 4.5.2 (R Foundation for Statistical Computing, Vienna, Austria) and Review Manager 5.4 (RevMan 5.4) software (Cochrane, London, UK). All meta-analyses were performed using a random-effects model based on the inverse variance method. Proportional outcomes were pooled using the Freeman–Tukey double arcsine transformation [39] and hazard ratios (HRs) were pooled using log-transformation. Between-study variance was estimated using the restricted maximum likelihood method [40]. Publication bias from small study effects were assessed using funnel plots and Egger’s test [41]. *p*-values < 0.05 were considered statistically significant. In forest plot figures, squares represent proportions based on individual studies and diamonds represent aggregated proportions. 

### 2.8. Subgroup and Sensitivity Analysis

Subgroup analysis included analysis based on treatment modality (e.g., chemotherapy, concurrent chemotherapy, sequential chemotherapy and radiotherapy).

## 3. Results

### 3.1. Literature Search Results

**Figure 1:**
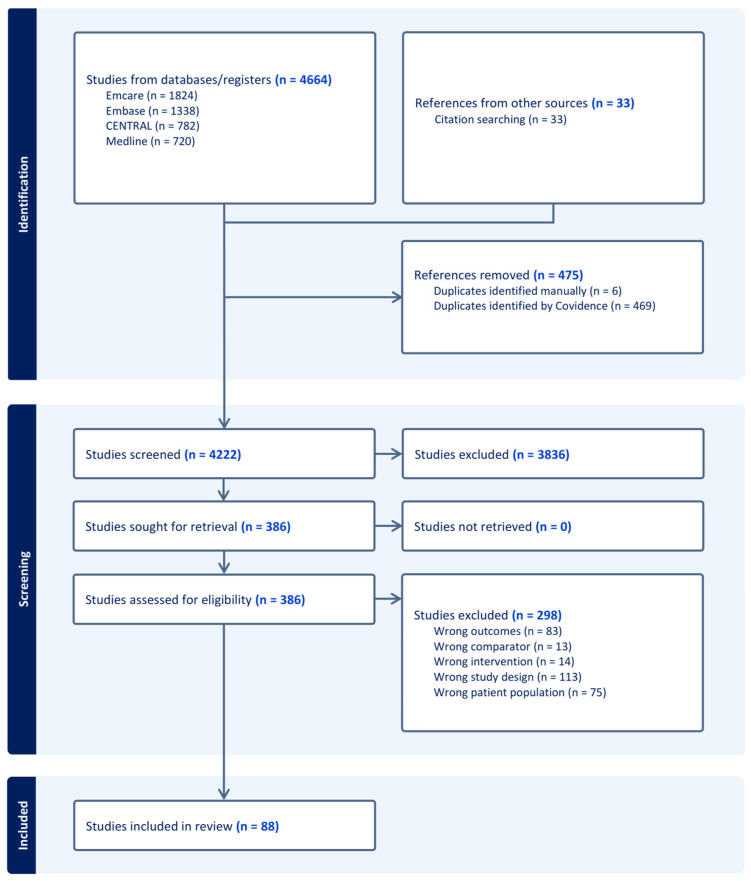
Search strategy and workflow in accordance with PRISMA guidelines.

The PRISMA flow diagram of the literature search is highlighted in Figure 1. Overall, 4664 articles were captured in a computer-assisted search, of which 475 duplicates were removed. An additional 33 articles were included through citation search. Then, 4222 articles were subsequently screened for abstract and title, which resulted in 3836 exclusions. The remaining 386 articles underwent full-text analysis for assessment of eligibility, which resulted in the inclusion of 88 articles in this review.

### 3.2. Overview of Included Studies

Eighty-eight studies involving 8585 patients were included in this review, comprising 2974 BRPC and 5611 LAPC patients (Table 1) [23,24,28,31,32,42,43,44,45,46,47,48,49,50,51,52,53,54,55,56,57,58,59,60,61,62,63,64,65,66,67,68,69,70,71,72,73,74,75,76,77,78,79,80,81,82,83,84,85,86,87,88,89,90,91,92,93,94,95,96,97,98,99,100,101,102,103,104,105,106,107,108,109,110,111,112,113,114,115,116,117,118,119,120,121,122,123,124]. Patient cohort sizes ranged from *n* = 7 to *n* = 723. Study types include retrospective studies (*n* = 50), prospective or clinical trials (*n* = 37) and case series (*n* = 1). Reference staging definition was primarily NCCN (*n* = 57). Treatment modalities explored include chemotherapy (*n* = 29), concurrent chemoradiotherapy (*n* = 16), sequential chemotherapy and chemoradiotherapy (*n* = 7), sequential chemotherapy and radiotherapy (*n* = 8), radiotherapy (*n* = 1), sequential chemotherapy and IRE (*n* = 4), targeted therapy (*n* = 3, with no molecular restrictions for cohort selection) and immunotherapy (*n* = 3). Of these, 10 studies explored multiple modalities that could be analysed separately. For the purposes of our analysis, cohorts from these studies will be separated and analysed according to specific treatment modality when possible to prevent duplicate analysis. Twenty-eight studies had heterogenous cohorts in terms of neoadjuvant therapy given, with data that could not be aggregated for the purposes of this review. A detailed version of the table of papers can be found in Table S1.

### 3.3. Outcomes of Downstaging to Surgical Resection Following Neoadjuvant Therapy

#### 3.3.1. Objective Response Rate

**Figure 2:**
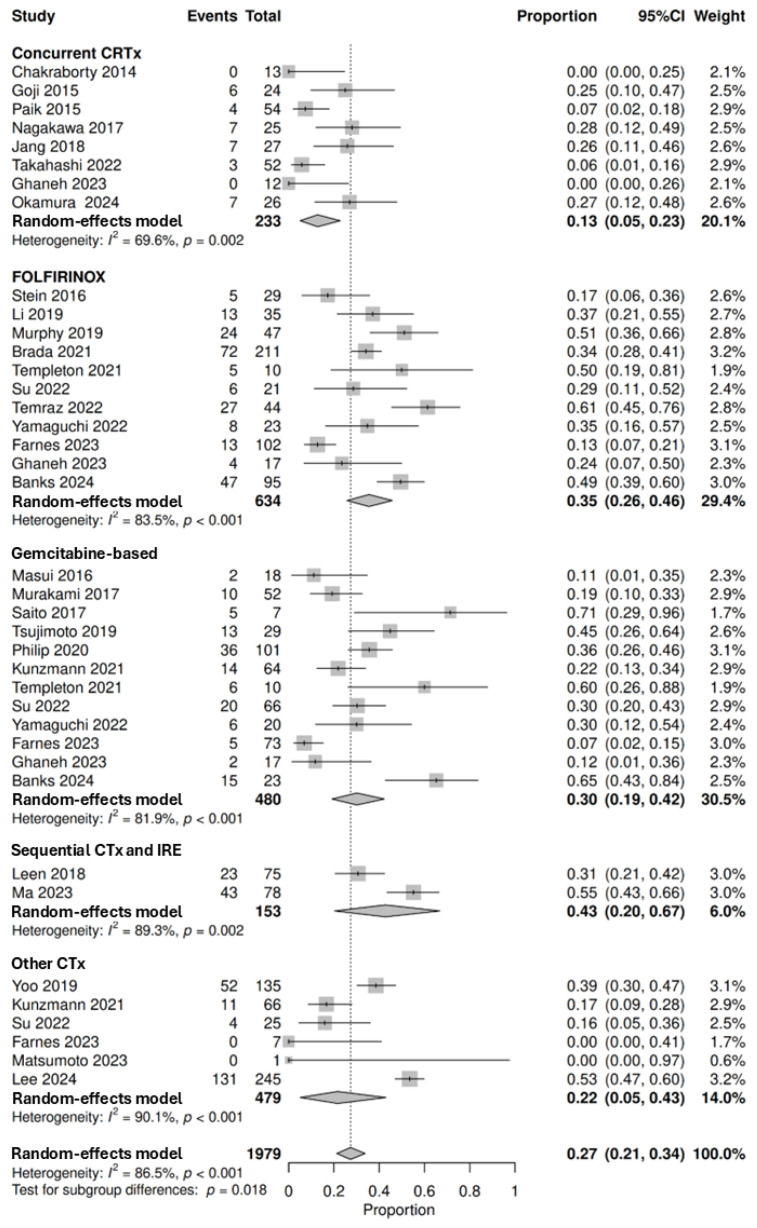
Forest plot denoting the proportion of patients that achieved a partial response or complete response following neoadjuvant therapies, including concurrent chemoradiotherapy (CRTx) [52,62,63,73,89,92,93,110], FOLFIRINOX [28,42,49,62,79,88,105,106,112,113,121], gemcitabine-based chemotherapy (CTx) regimens [28,42,62,75,82,87,97,101,106,112,116,121], sequential CTx and irreversible electroporation (IRE) [78,81] and other CTx regimens [42,75,77,83,106,123] (top-down). Objective response rate from studies were defined as per the RECIST criteria. Proportions span from 0 to 1.

The proportion of patients that achieved PR or CR as defined by the RECIST criteria is summarised in Figure 2. A total of 1979 patients from 29 studies were pooled for analysis, demonstrating that 27% (95% CI: 21–34%, with subgroup differences *p* = 0.018) achieved a PR or CR with neoadjuvant therapy. The proportion of patients that achieved PR or CR was highest in sequential chemotherapy and IRE at 43% (95% CI: 20–67%, *n* = 66/153 of which 8 patients achieved CR, 2 studies) followed by FOLFIRINOX at 35% (95% CI: 26–46%, *n* = 224/634 of which 10 patients achieved CR, 12 studies), gemcitabine-based regimens at 30% (95% CI: 19–42%, *n* = 134/480, 13 studies), other CTx regimens at 22% (95% CI: 5–43%, *n* = 198/479, 6 studies) and concurrent chemoradiotherapy at 13% (95% CI: 5–23%, *n* = 34/233 of which 1 patient achieved complete response, 7 studies). ORR following targeted therapies was explored in two studies [57,100]. Esnaola et al. reported that 18% (*n* = 6/34) achieved PR following cetuximab and chemotherapy. Sahora et al. reported that 17.86% (*n* = 5/28) achieved PR following bevacizumab and chemotherapy. ORR following immunotherapy was explored in two studies [31,81]. Du et al. reported that 60% (*n* = 15/25) achieved PR following PD-1 inhibitor and chemoradiotherapy. Ma et al. reported that 16% (*n* = 4/25) achieved CR and 48% (*n* = 12/25) achieved PR following combined treatment with PD-1/PDL-1 inhibitor, IRE and chemotherapy.

Some studies did not report a scoring system for objective response or used a modified tumour regression score. For concurrent chemoradiotherapy, He et al. reported CR and near-CR rates in 10% (*n* = 19/186) and 16% (*n* = 29/186) of patients, respectively [67]. Additionally, Amodeo et al. reported PR in 35% (*n* = 7/20) of patients [44]. For sequential chemotherapy and radiotherapy, Mellon et al. reported CR and PR rates in 7% (*n* = 4/58) and 41% (*n* = 24/58) of patients, respectively [85]. Furthermore, Zakem et al. reported CR, near-CR and PR rates in 7% (*n* = 5/73), 14% (*n* = 10/73) and 66% (*n* = 48/73) patients, respectively [124]. For sequential chemotherapy and IRE, Belfiore et al. reported PR in 90% (*n* = 18/20) of patients [46]. A further 11 studies could not be pooled for analysis (Table S2) [23,47,54,55,58,71,80,94,96,107,119].

#### 3.3.2. Proceeding to Surgery

**Figure 3:**
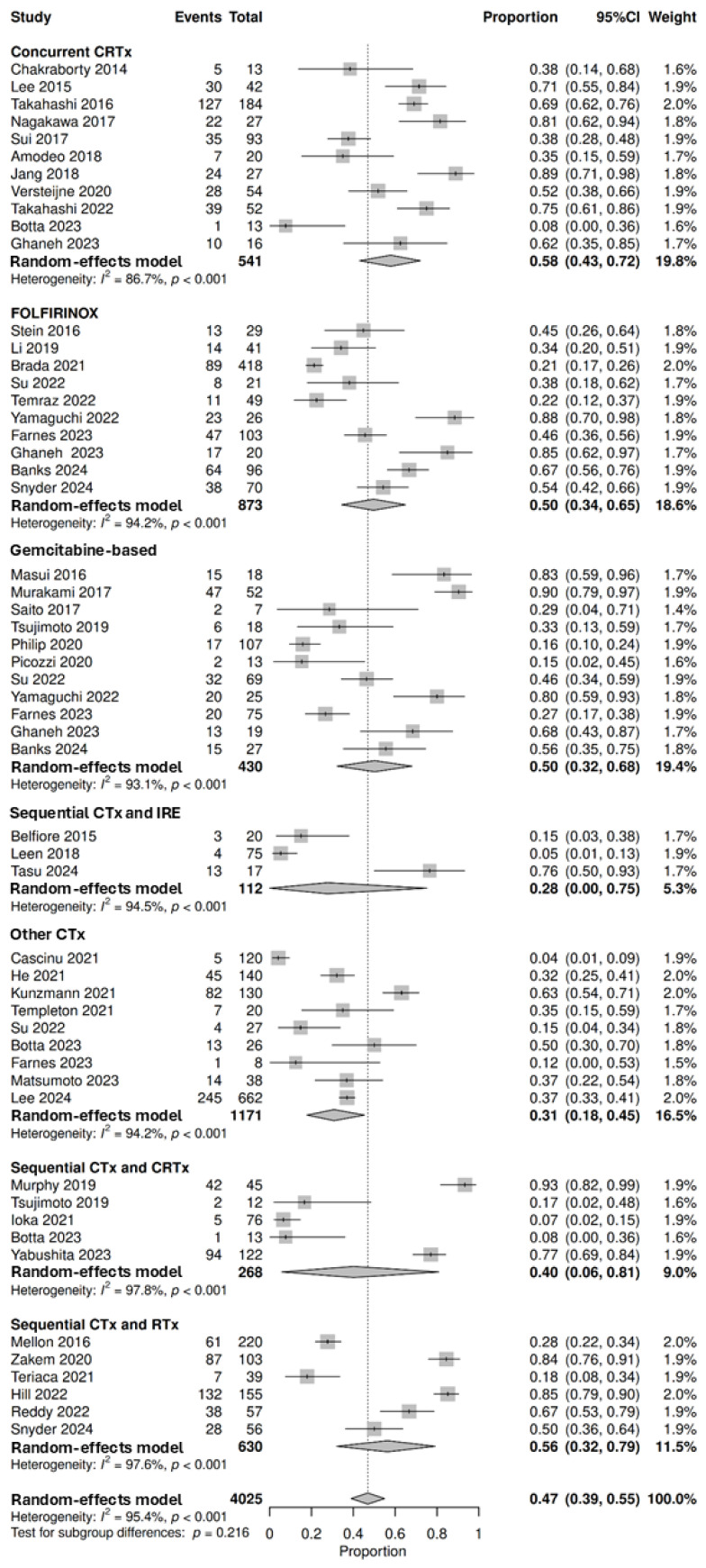
Forest plot denoting the proportion of patients that proceeded to surgery following neoadjuvant therapies, including concurrent chemoradiotherapy (CRTx) [44,48,52,62,73,76,89,108,109,110,117], FOLFIRINOX [28,42,49,62,79,104,105,106,113,121], gemcitabine-based chemotherapy (CTx) regimens [28,32,42,62,82,87,97,101,106,116,121], sequential chemotherapy and irreversible electroporation (IRE) [46,78,111], other CTx regimens [42,48,51,68,75,77,83,106,112], sequential CTx and CRTx [48,72,88,116,120], and sequential CTx and radiotherapy (RTx) [43,85,99,104,114,124] (top-down). Proportions span from 0 to 1.

A total of 43 neoadjuvant therapy studies that reported data based on intention to treat were included in this analysis, demonstrating that 47% (95% CI: 39–55%; no subgroup differences *p* = 0.216) of all enrolled participants (*n* = 4025) proceeded to surgery following neoadjuvant therapy. Rates of proceeding to surgery following neoadjuvant therapy were highest in patients treated with concurrent chemoradiotherapy at 58% (95% CI: 43–72%, *n* = 328/541, 11 studies), followed by sequential chemotherapy and radiotherapy at 56% (95% CI: 32–79%, *n* = 353/630, 6 studies), FOLFIRINOX at 50% (95% CI: 34–65%, *n* = 324/873, 10 studies), gemcitabine-based regimens at 50% (95% CI: 32–68%, *n* = 189/430, 11 studies), sequential chemotherapy and chemoradiotherapy at 40% (95% CI: 6–81%, *n* = 144/268, 5 studies), other chemotherapy regimens at 31% (95% CI: 18–45%, *n* = 416/1171, 9 studies) and sequential chemotherapy and IRE at 28% (95% CI: 0–75%, *n* = 20/112, 3 studies) (Figure 3). Most studies exploring targeted therapies or immunotherapy did not explicitly report rates of proceeding to surgery, and will be reported under resection rates. A further 28 studies could not be pooled for analysis (Table S3) [23,24,45,47,50,55,56,58,59,60,64,65,66,69,70,71,74,80,86,90,94,95,96,102,103,107,118,119].

#### 3.3.3. Rates of Resection

**Figure 4:**
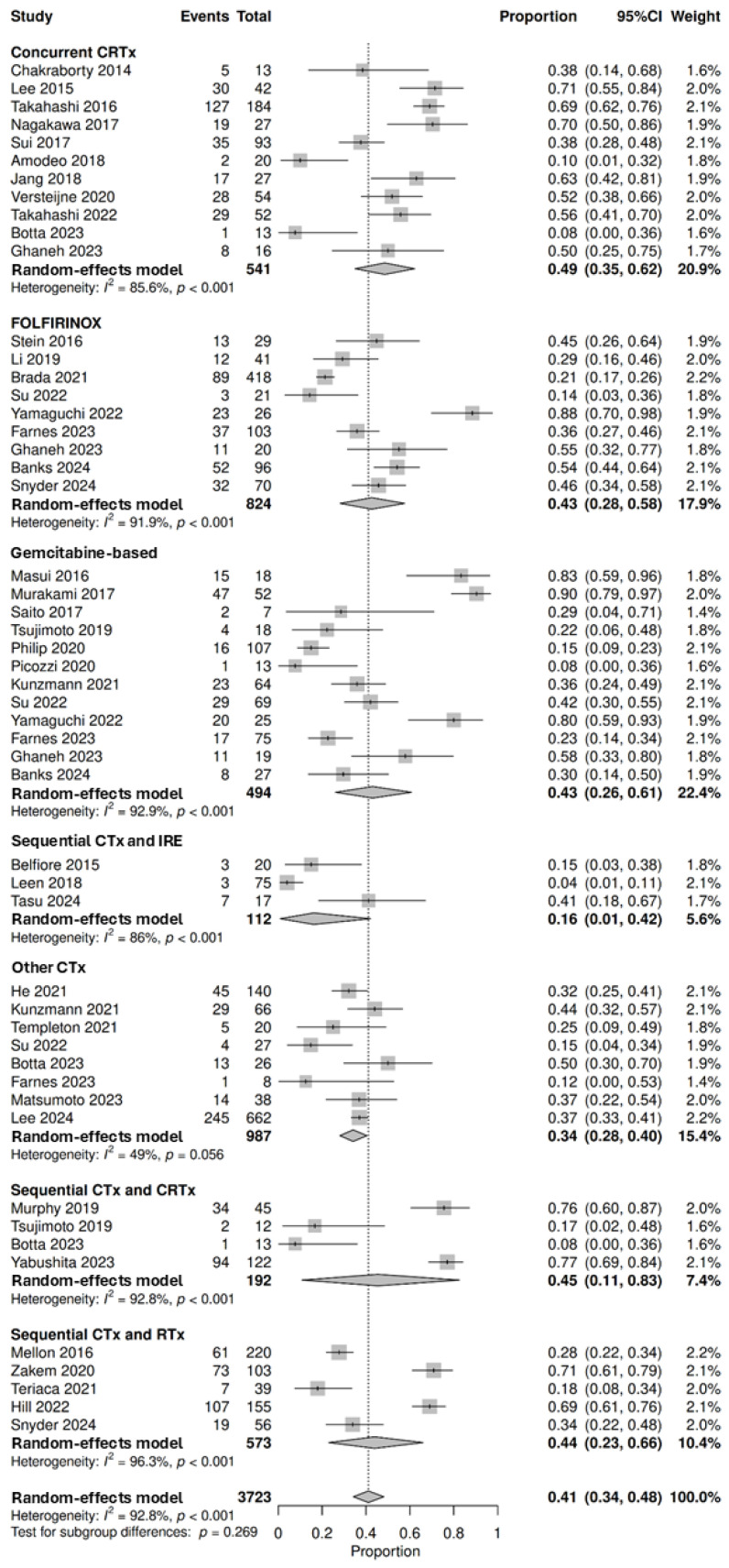
Forest plot denoting the proportion of patients that were resected following neoadjuvant therapies, including concurrent chemoradiotherapy (CRTx) [44,48,52,62,73,76,89,108,109,110,117], FOLFIRINOX [28,42,49,62,79,104,105,106,121], gemcitabine-based chemotherapy (CTx) regimens [28,32,42,62,75,82,87,97,101,106,116,121], sequential CTx and irreversible electroporation (IRE) [46,78,111], other CTx regimens [42,48,68,75,77,83,106,112], sequential CTx and CRTx [48,88,116,120], and sequential CTx and radiotherapy (RTx) [43,85,104,114,124] (top-down). Proportions span from 0 to 1.

To assess the rates of resection, 39 studies with intention to treat involving 3723 patients were pooled for analysis, demonstrating that 41% (95% CI: 34–48%; no subgroup differences *p* = 0.269) achieved curative-intent resection following neoadjuvant therapy. The highest resection rates were observed in patients treated with concurrent chemoradiotherapy at 49% (95% CI: 35–62%, *n* = 301/541, 11 studies), followed by sequential chemotherapy and chemoradiotherapy at 45% (95% CI: 11–83%, *n* = 131/192, 4 studies), sequential chemotherapy and radiotherapy at 44% (95% CI: 23–66%, *n* = 267/573, 5 studies), FOLFIRINOX alone at 43% (95% CI: 28–58%, *n* = 272/824, 9 studies), gemcitabine-based regimens at 43% (95% CI: 26–61%, *n* = 193/494, 12 studies), other chemotherapy regimens at 34% (95% CI: 28–40%, *n* = 356/987, 8 studies) and sequential chemotherapy and IRE at 16% (95% CI: 1–42%, *n* = 13/112, 3 studies) (Figure 4). Three studies explored resection rates following targeted therapy [32,57,100]. Picozzi et al. reported that 50% (*n* = 12/24) of patients underwent surgery following pamrevlumab and chemotherapy, with resection rates of 33% (*n* = 8/24). Esnaola et al. reported that 35% (*n* = 12/34) underwent surgery following cetuximab and chemotherapy, with further chemoradiotherapy in some cases, of which 32.35% (*n* = 11/34) were resected. Sahora et al. reported that 37% (*n* = 11/30) were resected following bevacizumab and chemotherapy. Two studies explored resection rates following immunotherapy [31,70] with Du et al. reporting that 40% (*n* = 10/25) of patients were resected following PD-1 inhibitor and chemoradiotherapy, while Hewitt et al. reported that 23% (*n* = 33/145) were resected following HAP-A immunotherapy vaccination and chemotherapy, with further chemoradiotherapy in some cases. The 28 studies that could not be pooled for analysis are described in Table S4 [23,24,45,47,50,55,56,58,59,60,64,65,66,69,70,71,74,80,86,90,94,95,96,102,103,107,118,119].

#### 3.3.4. Resection Status

**Figure 5:**
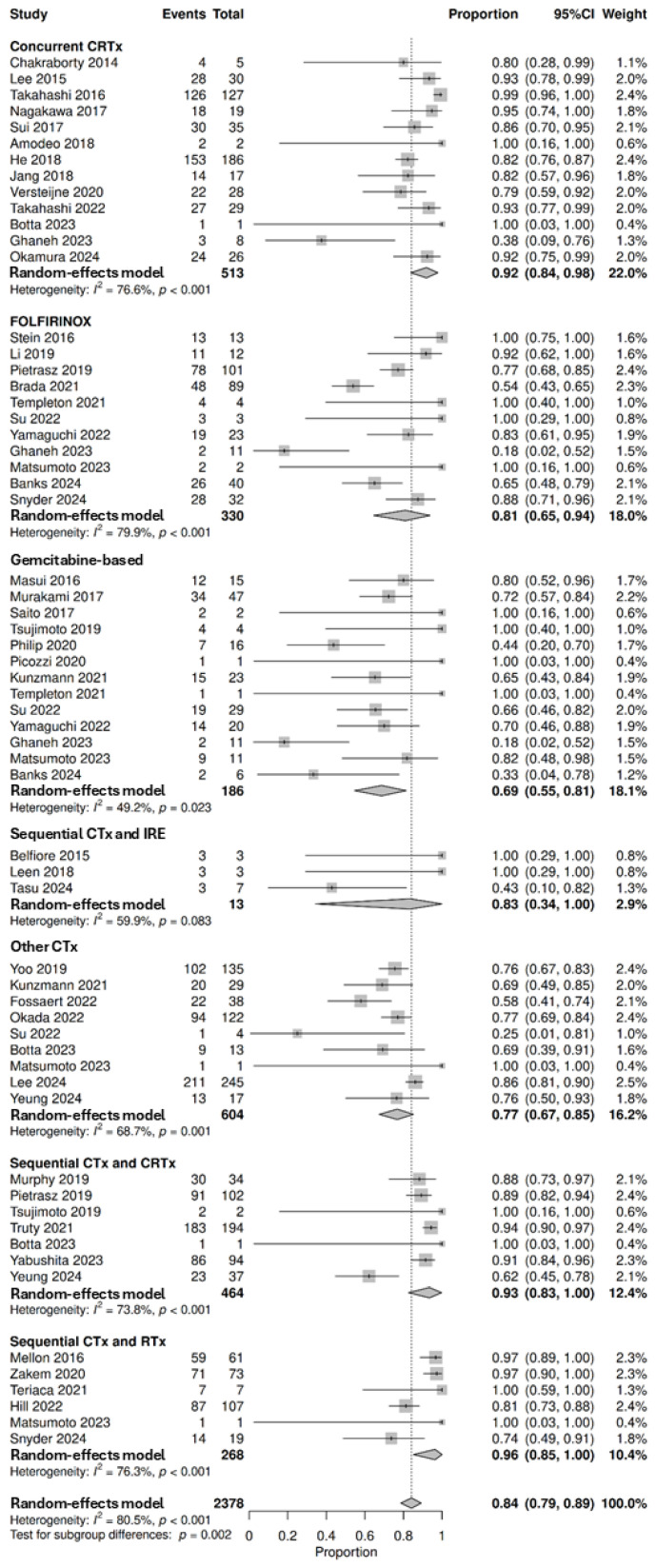
Forest plot denoting the proportion of patients that achieved R0 status after resection following neoadjuvant therapies, including concurrent chemoradiotherapy (CRTx) [44,48,52,62,67,73,77,89,92,108,109,110,117], FOLFIRINOX [28,49,62,79,83,98,104,105,106,112,121], gemcitabine-based chemotherapy (CTx) regimens [28,32,62,75,82,83,87,97,101,106,112,116,121], sequential CTx and irreversible electroporation (IRE) [46,78,111], other CTx regimens [48,61,75,77,83,91,106,122,123], sequential CTx and CRTx [48,88,98,115,116,120,122], and sequential CTx and radiotherapy (RTx) [43,83,85,104,114,124] (top-down). Proportions span from 0 to 1.

A total of 45 studies involving 2378 patients that underwent curative resection were pooled to analyse pathological resection outcome status, demonstrating that 84% (95% CI: 79–89%, with subgroup differences *p* = 0.002) achieved R0 resection following neoadjuvant therapy. The highest proportion of patients with R0 resections was after sequential chemotherapy and radiotherapy at 96% (95% CI: 85–100%, *n* = 239/268, 6 studies), followed by sequential chemotherapy and chemoradiotherapy at 93% (95% CI: 83–100%, *n* = 416/464, 7 studies), concurrent chemoradiotherapy at 92% (95% CI: 84–98%, *n* = 452/513, 13 studies), sequential chemotherapy and IRE at 83% (95% CI: 34–100%, *n* = 9/13, 3 studies), FOLFIRINOX at 81% (95% CI: 65–94%, *n* = 234/330, 11 studies), other chemotherapy regimens at 77% (95% CI: 67–85%, *n* = 473/604, 9 studies) and gemcitabine-based regimens at 69% (95% CI: 55–81%, *n* = 122/186, 13 studies) (Figure 5). Only one study by Chen et al. explored radiotherapy alone; however, the study did not report resection status [53]. Three studies explored resection status following targeted therapy [32,57,100]. Picozzi et al. observed that 50% (*n* = 4/8) of patients achieved R0 following pamrevlumab and chemotherapy. Esnaola et al. observed an R0 rate of 100% (*n* = 11/11) following cetuximab and chemotherapy, with further chemoradiotherapy in some cases. Sahora et al. observed that 91% (*n* = 10/11) of patients achieved R0 following bevacizumab and chemotherapy. One study by Du et al. explored resection status following immunotherapy, and reported an R0 rate of 90% following PD-1 inhibitor and chemoradiotherapy [31]. A further 28 studies could not be pooled for analysis (Table S4) [23,24,45,47,50,55,56,58,59,60,64,65,66,69,70,71,74,80,86,90,94,95,96,102,103,107,118,119].

#### 3.3.5. Nodal Status

**Figure 6:**
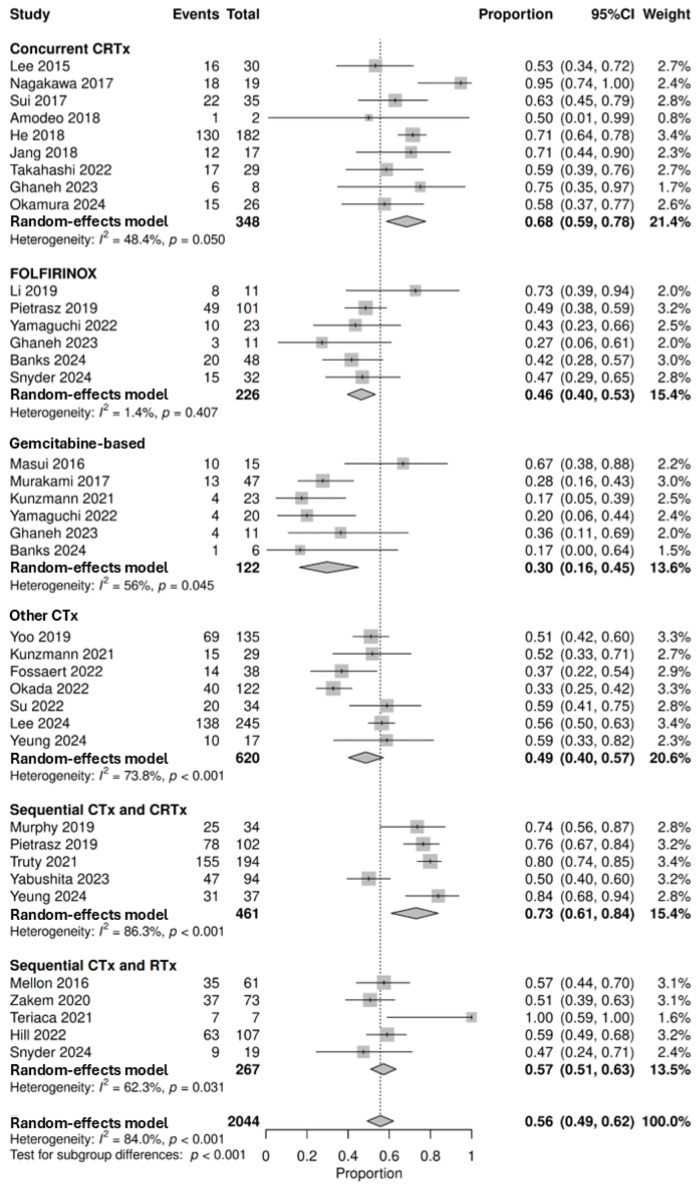
Forest plot denoting the proportion of patients that achieved N0 status after resection following neoadjuvant therapies, including concurrent chemoradiotherapy (CRTx) [44,62,67,73,76,89,92,108,110], FOLFIRINOX [28,62,79,98,104,121], gemcitabine-based chemotherapy (CTx) regimens [28,62,75,82,87,121], other CTx regimens [61,75,77,91,106,122,123], sequential CTx and CRTx [88,98,115,120,122], and sequential CTx and radiotherapy (RTx) [43,85,104,114,124] (top-down). Proportions span from 0 to 1.

To assess the impact of neoadjuvant therapy on pathological nodal status, 30 studies involving 2044 patients that underwent curative resection were pooled for analysis, demonstrating that 56% (95% CI: 49–62%, with subgroup differences *p* < 0.001) achieved N0 status with neoadjuvant therapy. The proportion of patients with N0 status was highest following sequential chemotherapy and chemoradiotherapy at 73% (95% CI: 61–84%, *n* = 336/461, 5 studies) followed by concurrent chemoradiotherapy at 68% (95% CI: 59–78%, *n* = 237/348, 9 studies), sequential chemotherapy and radiotherapy at 57% (95% CI: 51–63%, *n* = 151/267, 5 studies), other chemotherapy regimens at 49% (95% CI: 40–57%, *n* = 306/620, 7 studies), FOLFIRINOX at 46% (95% CI: 40–53%, *n* = 105/226, 6 studies) and gemcitabine-based regimens at 30% (95% CI: 16–45%, *n* = 36/122, 6 studies) (Figure 6). One study by Chen et al. found that 54% (*n* = 53/98) achieved N0 following radiotherapy and resection [53]. No studies reported nodal status following neoadjuvant IRE, targeted therapy, or immunotherapy. A further 16 studies could not be pooled for analysis (Table S5) [23,24,56,58,64,66,69,71,80,83,86,94,95,96,102,119].

### 3.4. Oncological Outcomes

#### 3.4.1. Overall Survival

**Figure 7:**
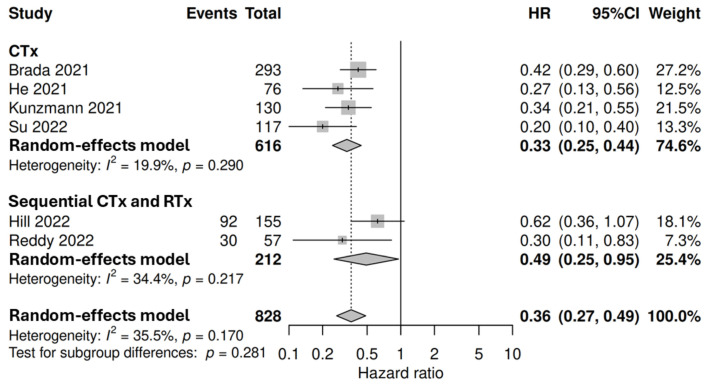
Forest plot of overall survival hazard ratios following neoadjuvant therapy with or without resection, including chemotherapy (CTx) [49,68,75,106] and sequential CTx and radiotherapy (RTx) [43,99]. Hazard ratios < 1 represents OS benefit. The number of events denotes the number of patients that were reported to have died, and was recorded if available.

There was heterogeneous reporting of OS data. Only six studies had sufficient data for pooled analysis of OS HRs for patients that received neoadjuvant therapy with resection compared to without resection. This analysis of 828 patients demonstrated improved outcomes for patients treated with neoadjuvant therapy and resection compared to non-operative management (HR 0.36, 95% CI 0.27–0.49), with a resection rate of 44% (*n* = 365/828). Improved OS was demonstrated for those resected following both neoadjuvant chemotherapy (HR 0.33, 95% CI 0.25–0.44, *n* = 616, 4 studies) and neoadjuvant sequential chemotherapy and radiotherapy (HR 0.49, 95% CI 0.25–0.95, *n* = 212, 2 studies) with resection rates of 36% (*n* = 220/616) and 68% (*n* = 145/212) respectively. However no significant differences in HRs were demonstrated between modalities (*p* = 0.281) (Figure 7).

Overall, a majority of studies reported OS in months, and were deemed too heterogenous for pooled analysis. Fourteen studies involving 1459 patients evaluated OS for patients who underwent neoadjuvant chemotherapy with (*n* = 810) or without (*n* = 649) resection [49,61,68,75,77,79,82,87,91,98,106,112,122,123]. OS for those resected ranged from 7.5 months (for R1 resections) to 43.2 months (95% CI: 38.4–48.1), compared to without resection ranging from 8.7 months (95% CI: 4.4–22) to 21.5 months (95%: CI 19.7–23.3) (*p* < 0.05 in six out of seven studies) (Table S6). Neoadjuvant concurrent chemoradiotherapy was compared for OS in five studies involving 167 patients with (*n* = 106) or without resection (*n* = 61) [44,76,89,92,110]. OS for those resected ranged from 22.9 months to 41.8 months (95% CI: 27–56.6) compared to without resection, ranging from 9.3 months to 19.5 months (*p* < 0.05 in two out of four studies) (Table S7). Neoadjuvant sequential chemotherapy and chemoradiotherapy was reported for five studies involving 489 patients with (*n* = 461) or without resection (*n* = 28). OS following sequential chemotherapy and chemoradiation with resection ranged from 23 months to 57.8 months (95% CI: 42.6–73). One study additionally compared neoadjuvant sequential chemotherapy and chemoradiotherapy with resection (OS = 24.7 months, *n* = 94) compared to without resection (OS = 9.6 months, *n* = 28) (*p* < 0.001) (Table S8) [88,98,115,120,122]. Neoadjuvant sequential chemotherapy and radiotherapy was evaluated in five studies involving 513 patients with (*n* = 286) or without (*n* = 227) resection [43,84,99,114,124]. OS for those resected ranged from 24 months (95% CI: 21.6–26.3) to 34.2 months, compared to unresected cohorts ranging from 7 months to 17 months (*p* < 0.05 in 5/5 studies) (Table S9). Novel treatments including IRE, targeted therapy and immunotherapy were evaluated in four studies. Tasu et al. reported improved OS for patients who underwent IRE with sequential chemotherapy and resection (OS = 31 months, 95% CI: 4–undefined) versus those without resection (OS = 21 months, 95% CI: 4–25) (*p* = 0.044) [111]. Esnaola et al. found that those with R0 resections following cetuximab and chemotherapy, with or without additional chemoradiotherapy, had improved survival (median OS not yet reached) [57]. Meanwhile, Sahora et al. demonstrated no difference in survival (resected OS = 13 months, 95% CI: 10.8–15.2; unresected OS = 13 months, 95% CI:11.6–14.4, *p* = 0.131) following neoadjuvant chemotherapy with concurrent bevacizumab [100]. For the use of sequential HAP-A immunotherapy vaccination with chemotherapy, with or without further chemoradiotherapy, OS was 27.1 months compared to 12.6 months in the unresected cohort (no *p*-value given) [70]. A further 17 studies could not be pooled for analysis (Table S10) [23,24,45,50,59,64,65,69,70,71,80,86,95,102,103,118,119].

#### 3.4.2. Recurrence-Free Survival

RFS or disease-free survival (DFS) was heterogeneously reported and could not be pooled for analysis. Following neoadjuvant chemotherapy and resection reported, six studies involving 568 patients reported either RFS or DFS ranges between 9.0 months (95% CI: 6.8–11.2) and 22.7 (95% CI: 17.9–27.5) for LAPC or 26.0 months (95% CI: 21.7–30.3) for BRPC patients (Table S11) [61,77,82,98,106,123]. Okamura et al. reported RFS of 11 months (*n* = 26) after resection following neoadjuvant concurrent chemoradiotherapy [92]. Additionally, He et al. reported higher DFS with pathological complete responses (26 months, *n* = 18) compared to near-complete response (12 months, *n* = 29) or limited response (12 months, *n* = 135) after resection following neoadjuvant concurrent chemoradiotherapy [67] (Table S12). The use of sequential chemotherapy and chemoradiotherapy in three studies involving 390 patients with curative-intent resection demonstrated RFS or DFS ranging from 17.5 months to 23.5 months (Table S13) [98,115,120]. There were no studies that reported RFS or DFS after resection following neoadjuvant sequential chemotherapy and radiotherapy, IRE, targeted therapy or immunotherapy. A further nine studies listed in Table S14 could not be pooled for analysis (Table S14) [23,24,50,59,64,80,96,102,103].

#### 3.4.3. Progression-Free Survival

PFS could not be pooled for analysis due to heterogenous reporting. The impact of neoadjuvant chemotherapy on PFS was evaluated in five studies involving 387 patients with (*n* = 231) or without (*n* = 156) resection [68,79,106,112,123]. The use of neoadjuvant chemotherapy followed by resection was associated with a PFS ranging from 10.6 months to 19.3 months, compared to without resection ranging from 5.7 months (95% CI 3.8–7.7) to 11.9 months (*p* < 0.05 in three out of four studies) (Table S15). The use of neoadjuvant concurrent chemoradiotherapy in three studies involving 68 patients with (*n* = 60) or without (*n* = 8) resection demonstrated an associated PFS ranging from 12.5 months to 21.6 months (10.9–40.9), compared to 2.1 months (95% CI 1.5–2.4 months) without resection (no *p*-values given) [52,92,110] (Table S16). Neoadjuvant sequential chemotherapy and radiotherapy evaluated in 216 patients across two studies with (*n* = 99) or without (*n* = 117) resection was associated with a PFS of 12.9 months to 23.1 months, compared to 1.6 months to 9.5 months without resection (*p* < 0.05 in two out of two studies) [84,99] (Table S17). Furthermore, the use of neoadjuvant sequential chemotherapy and radiotherapy followed by resection has been demonstrated to achieve a local PFS of 27.7 months [43]. Individual studies have explored the impact of neoadjuvant sequential chemotherapy and chemoradiotherapy followed by resection, demonstrating a PFS of 21.3 months (95% CI: 16.6–28.2) [88] and the role of immunotherapy (PD-1 inhibitor) with concurrent chemoradiotherapy, highlighting a PFS of 28.23 months for those who achieved an R0 resection compared with 10.62 months for those with R1 resection or no resection [31]. There were no studies that reported PFS following neoadjuvant IRE or targeted therapy. A further four studies are listed in Table S18 but could not be pooled for analysis (Table S18) [45,47,71,119].

### 3.5. Evaluation of Risk-of-Bias and Quality Assessment

Fifty-four studies were included in meta-analyses and analysed for risk of bias and quality. Based on the RoB 2, three studies [32,62,75] were assessed as low risk of bias and five studies [51,72,73,104,117] had some concerns (Figure S1). Based on the MINORS, 7 studies [63,97,110,111,113,120,121] were assessed as high quality, 16 studies [43,44,46,52,78,79,88,89,92,93,99,105,108,109,114,116] as moderate quality and 1 study [101] as low quality (Table S19). Based on the ROBINS-I, 13 studies [28,42,48,49,68,76,81,82,83,87,106,112,124] were assessed as moderate risk of bias and 9 studies [61,67,77,85,91,98,115,122,123] as high risk of bias (Figure S2). Studies considered as having serious risk of bias retrospectively included only patients that were resected following neoadjuvant therapy. For the purposes of our study, if the unresected cohorts were not reported in these studies, they will be removed from the proceeding-to-surgery and resection rate analyses. Funnel plots and Egger’s test were generated for all outcomes except for OS due to the limited number of studies. All results demonstrated no significant publication bias from small study effects (all *p* > 0.05) (Figures S3–S7). Overall, the certainty of evidence was considered low, except for OS, which was graded as moderate, mainly due to potential risk of bias and clinical heterogeneity of studies (Table 2).

## 4. Discussion

This updated systematic review and meta-analysis provides contemporary insights into how different neoadjuvant strategies influence downstaging, surgical and oncological outcomes for patients with BRPC and LAPC. Across the studies evaluated in pooled analyses, we demonstrated that multimodal regimens incorporating both chemotherapy and radiotherapy, whether concurrent or sequential, had the most optimal surgical outcomes with the highest proportions of R0 and N0 status after curative resection. Overall, patients who underwent either neoadjuvant chemotherapy alone and sequential chemotherapy and radiotherapy with curative-intent resection had a substantial survival advantage compared to those who were managed non-operatively or failed to be resected. However, there were no significant differences in survival benefit between these two modalities. Other neoadjuvant modalities could not be compared for analysis given the paucity of data that could be pooled. These outcomes arise from highly heterogenous studies with potential risk of bias, and were made with low certainty, with the exception of overall survival benefit, which was made with moderate certainty based on our GRADE assessment. However, when taken together, our review reinforces the evidence for combining chemotherapy and radiotherapy modalities, whether concurrent or sequential in the neoadjuvant setting, to improve the quality of resection for patients with BRPC and LAPC and posits that neoadjuvant approaches (namely chemotherapy alone or sequential chemotherapy and radiotherapy) with resection have overall survival advantages compared to non-operative management with the same modalities.

This review also highlights modality-specific insights that may guide further research and refinement of neoadjuvant therapy guidelines. When considering regimens with chemotherapy alone, there was wide variability in regimens and outcomes. In general, FOLFIRINOX had higher outcomes compared to gemcitabine-based regimens, either as monotherapy, doublets or rarely quartets. The performance of neoadjuvant chemotherapy, although outperforming chemoradiotherapy in terms of ORR, had more modest results for margin-negative resection and N0 status. The addition of radiotherapy, whether combined or sequential with chemotherapy, demonstrated more consistent results, with higher rates of R0 resection and N0 status, indicating more successful downstaging and improved locoregional control of BRPC and LAPC. This is largely congruent with previous reviews that report higher rates of R0 with chemoradiotherapy or the addition of radiotherapy to chemotherapy for patients with resectable or BRPC [125,126]. When we evaluated IRE, the evidence suggested that this modality is predominantly used for the control of unresectable disease, rather than a neoadjuvant conversion strategy [127,128]. Although our analysis demonstrated that sequential chemotherapy and IRE had the greatest ORR, there remains a paucity of evidence with high variability and low strength of evidence surrounding the efficacy and utility of neoadjuvant IRE compared to other more conventional modalities. This limits conclusions drawn from our analysis, and highlights the need for robust prospective trial or registry data to provide insights into neoadjuvant IRE. Finally, it is difficult to draw conclusions for biologics and immunotherapy given the paucity of data and potential confounders since these studies variably incorporated mixtures of additional chemotherapy and chemoradiotherapy. This prevents pooled analysis, and further highlights the need for future studies for these modalities.

The current approach to neoadjuvant therapy for BRPC and LAPC remains an area of ongoing debate. In particular, there is a lack of consensus surrounding the ideal patient for neoadjuvant therapy, the ideal modality, duration, dose and regimen of neoadjuvant therapy, and the ideal time to surgical resection once adequately downstaged. Emerging evidence suggests that an overall strategy of neoadjuvant therapy incorporating strategies for systemic and local control for downstaging and subsequent resection leads to improved outcomes for BRPC and LAPC. The recent REDISCOVER consensus conference put forward 30 recommendations for best-practice strategies in navigating treatment for patients with BRPC and LAPC [129]. Central and relevant to this discussion are recommendations to indeed resect tumours following neoadjuvant therapy, and to resect them as soon as safe and practical. However, this management approach proves difficult for the following reasons. Contextually, the lack of standardised definitions of BRPC and LAPC may result in variable decision-making for surgical eligibility [130]. This can often result in subjective outcomes and additionally limits the translatability of evidence into the clinical setting. Radiological evaluation, a pejorative factor in evaluating surgical eligibility, may also overestimate tumour responses, or may not detect satellite lesions and other occult metastasis that may result in surgical abandonment [4,18]. This highlights a separate issue as there is a need for more sensitive radiological approaches that can more accurately diagnose pancreatic cancer and subsequent tumour responses and to diagnose disease at an earlier stage to improve management outcomes [131]. In addition, for patients with optimal responses to neoadjuvant treatment, the option of surgery may also be limited based on the skillset of the surgeon and hospital facilities, with guidelines supporting centralisation of surgical oncology resources for quality [129]. These are all reasons that may highlight the lack of congruency between optimal tumour response and proceeding to surgery and resection. Overall, our findings, as well as those of the REDISCOVER consensus committee, suggest there is a need for more prospective trials or development of prospective registries that will allow quantitative comparison to more robustly evaluate downstaging, resectional and oncological outcomes of different neoadjuvant treatment modalities to better define a standard of treatment.

Overall, this study provides a comprehensive review of current and novel modalities for the management of BRPC and LAPC in the past decade. However, there are important limitations to note with our review. Overall, there are significant biases that should be recognised. The significant heterogeneity of BRPC and LAPC definitions and cohorts, which were not accounted for in our analysis, may introduce classification bias and cohort selection bias and limit the comparability of studies. There is also significant clinical heterogeneity amongst included studies, such as variations in regimens, dosing and duration across and within modalities, and type of radiotherapy used, which prevented further subgroup analyses that could provide further insight. Alongside this, resection may be variably offered depending on the tertiary centre and skillset of the surgeon, which can introduce publication bias in outcomes including resection rates [129]. Furthermore, the studies included were predominately retrospective (57%, *n* = 50), which may introduce selection and recall biases or other potential confounders. In addition, this review did not encompass biological, tumour and patient factors that may influence downstaging, resection rates and subsequent oncological outcomes that are unrelated to and may impact the type of neoadjuvant modality used. For example, the normalisation of Ca-19.9 levels may be an additional indicator of ‘biological’ downstaging following neoadjuvant treatment that can guide surgical reassessment [132]. Furthermore, tumour factors such as arterial involvement typically result in poorer surgical and survival outcomes compared to venous and can confound outcomes, whilst other patient factors including age and morbidity status may also influence the suitability of specific neoadjuvant modalities offered and whether a patient is fit for surgery [4,109,129]. Altogether, these limitations reflect the clinical heterogeneity of the current literature and the lack of a standardised definition, approach and guidelines for managing BRPC and LAPC. Although this limits the certainty of evidence and clinical applicability of current evidence from our analysis, these factors highlight the need for continual multidisciplinary and multimodal approaches to identify patients suitable for neoadjuvant downstaging and surgical reassessment, with more robust clinical trials or prospective registries that account for these important variables and biases [129,133].

## 5. Conclusions

There is growing evidence for downstaging BRPC and now even LAPC to resection with neoadjuvant therapy, aimed to improve oncological outcomes. This review highlights current and novel modalities for management in this way, reporting promising resection outcomes following regimens that incorporated both chemotherapy and radiotherapy, either concurrent or sequential. Despite this, there were no subgroup differences in OS benefit in our limited analysis of chemotherapy alone versus sequential chemotherapy and radiotherapy. Further conclusions about survival benefit cannot be made for other regimens given the clinical heterogeneity of current data. More clinical trials are warranted to explore and compare these modalities to determine optimal treatment strategies for BRPC and LAPC.

## Figures and Tables

**Table 1:** Table of included studies.

Author (Year)	Country of Publication	Age (Mean or Median; Range)	Total Patients (*n*)	BRPC:LAPC	Reference Definition	Treatment Modality
Prospective studies						
Amodeo et al. (2018) [44]	USA	64.5 (46–76)	24	13:11	NCCN	Concurrent CRTx
Belfiore et al. (2015) [46]	Italy	69.2 (55–82)	20	0:20	NCCN	Sequential IRE and CTx
Cascinu et al. (2021) [51]	Italy	Group 1: 72 (42–79) Group 2: 68 (36–74)	124	0:124	NCCN	CTx
Chakraborty et al. (2014) [52]	USA	66 (51–82)	13	13:0	MDACC	Concurrent CRTx
Du et al. (2023) [31]	China	62 (40–75)	25	10:15	NCCN	Concurrent immunotherapy with CRTx
Esnaola et al. (2014) [57]	USA	60 (28–78)	37	13:24	NCCN	Concurrent biologic with CTx, some with sequential CRTx
Farnes et al. (2023) [42]	Norway	69.5 (63–74)	230 (188 analysable)	96:92	NCCN	CTx
Fietkau et al. (2021) [59]	Germany	66 (41–79)	180	0:180	NR	CTx, some with sequential CRTx
Flak et al. (2019) [60]	Denmark	67.1 (50–81)	33	0:33	NCCN	IRE with or without sequential CTx or RTx
Ghaneh et al. (2023) [62]	UK, Germany	63 (57–69)	86 (55 analysable)	55:0	NCCN	CTx or concurrent CRTx
Goji et al. (2015) [63]	Japan	68 (47–81)	25	0:25	NR	Concurrent CRTx
Hammel et al. (2016) [65]	France, Australia, Belgium, Sweden	63.3	442	0:442	International Union Against Cancer staging system	CT or concurrent CRTx with or without concurrent biologic
Herman et al. (2015) [69]	USA	67 (35–87)	49	0:49	AHPBA/SSO/GSSC	RTx, some with sequential CTx
Hewitt et al. (2022) [70]	USA	NR	303	53:250	NCCN	CTx with or without sequential CRTx, some with cancer vaccine immunotherapy
Ioka et al. (2021) [72]	Japan	Arm A: 65 (38–78) Arm B: 67 (28–80)	100	0:100	UICC	CRTx with or without induction CTx
Jang et al. (2018) [73]	South Korea	59.4 ± 8.4	27	27:0	NCCN	Concurrent CRTx
Kunzmann et al. (2021) [75]	Germany	Group 1: 61 (55–67) Group 2: 63.5 (56–69)	130	0:130	NCCN	CTx
Li et al. (2019) [79]	China	62 (44–80)	41	0:41	NCCN	CTx
Masui et al. (2016) [82]	Japan	63 (43–73)	18	18:0	NCCN	CTx
Moningi et al. (2015) [86]	USA	67.1 (35.6–87.5)	88	14:74	AHPBA/SSO/SSAT	RTx, some with sequential CTx
Murphy et al. (2019) [88]	USA	63 (42–78)	49	0:49	NCCN	CTx, with sequential CRTx
Nagakawa et al. (2017) [89]	Japan	65.1 (36–76)	27	27:0	NCCN	Concurrent CRTx
Philip et al. (2020) [97]	USA, France, Italy, Spain, Canada	65.0 (60.0–72.0)	107	0:107	AHPBA/SSO/SSAT	CTx
Picozzi et al. (2020) [32]	USA	66	37	0:37	NCCN	CTx, some concurrent with biologic
Sahora et al. (2014) [100]	Austria	62 (43–80)	32 (30 analysable)	11:19	NCCN	Concurrent biologic with CTx
Snyder et al. (2024) [104]	USA	63 (39–80)	126 (51 analysable)	51:0	NCCN	CTx, some sequential with RTx
Stein et al. (2016) [105]	USA	63 (46–79)	31	11:20	NCCN	CTx
Sudo et al. (2017) [107]	Japan	67.5 (44–79)	30	0:30	NR	CTx, some with sequential CRTx and further CTx
Takahashi et al. (2016) [109]	Japan	NR	184	184:0	NCCN	Concurrent CRTx
Takahashi et al. (2022) [110]	Japan	66 (48–75)	52	52:0	NCCN	Concurrent CRTx
Tasu et al. (2024) [111]	France	61 (37–77)	17	0:17	NCCN	Sequential IRE with CTx
Temraz et al. (2022) [113]	Lebanon	61.8 ± 7.9	49	20:29	NCCN	CTx
Teriaca et al. (2021) [114]	The Netherlands	63 (40–74)	50	0:50	DPCG	Sequential CTx and RTx
Versteijne et al. (2020) [117]	The Netherlands	NR	54	54:0	UICC	Concurrent CRTx
Weisz Ejlsmark et al. (2024) [118]	Denmark	NR	28	0:28	NCCN	RTx, some with sequential CTx
Yabushita et al. (2023) [120]	Japan	70 (37–89)	122	122:0	NCCN	Concurrent CRTx
Yamaguchi et al. (2022) [121]	Japan	Group 1: 66 (58–73) Group 2: 66 (58–71)	51	51:0	NCCN	CTx
Retrospective studies						
Banks et al. (2024) [28]	Australia	NEO: <60 *n* = 46, ≥60 *n* = 82	152 (128 analysable)	128:0	NR	CTx, CRTx or RTx
Bednar et al. (2017) [45]	USA	66.5 (36–86)	92	0:92	NCCN	CTx, some sequential with RTx
Blazer et al. (2015) [47]	USA	62.4 (40–81)	43	18:25	AHPBA/SSAT/SSO/GSSC	CTx or concurrent CRTx
Botta et al. (2023) [48]	USA	70.6 (44–87)	52	33:19	NCCN	CTx, CRTx or sequential CTx and CRTx
Brada et al. (2021) [49]	The Netherlands	Resected: 62 ± 9 Unresected: 62 ± 9	418 (293 analysable)	0:293	DPCG	CTx
Brada et al. (2025) [50]	The Netherlands	66 (57–69)	103	0:103	DPCG	CTx or concurrent CRTx
Chen et al. (2017) [53]	China	<60 45 (45.9%) >60 53 (54.1%)	98	0:98	AJCC 6th or 7th edition	RTx
Chen et al. (2022) [54]	Taiwan	63 (41–85)	57	0:57	NR	RTx, some with sequential CTx or concurrent CRTx
Comito et al. (2023) [55]	Italy	71 (41–91)	142	0:142	AHPBA/SSO/SSAT	RTx, some with sequential CTx
Dai et al. (2023) [56]	Canada	65.8 ± 9.3	723	0:723	NR	CTx, some with prior RTx or surgery
Ferrone et al. (2015) [58]	USA	62 (38–77)	40	15:25	AHPBA/SSO/SSAT	CTx or concurrent CRTx
Fossaert et al. (2022) [61]	France	66	38	38:0	MDACC	CTx
Gemenetzis et al. (2019) [23]	USA	65.2 ± 10.1	415	0:415	NCCN	CTx, some with sequential RTx
Groot et al. (2019) [64]	USA	62.7 ± 9.4	231	138:93	NCCN	CTx or concurrent CRTx
Han et al. (2020) [66]	South Korea	62.1 ± 10.8	26	26:0	NCCN	CTx or concurrent CRTx or RTx
He et al. (2018) [67]	USA	NR	186	87:99	NR	Concurrent CRTx
He et al. (2021) [68]	China	Group 1: 60 (39–80) Group 2: 59 (39–70) Group 3: 59 (34–87)	140	0:140	NCCN	CTx with either conversion surgery or IRE
Hill et al. (2022) [43]	USA	66 (42–84)	155	91:64	NCCN	CTx, with sequential RTx
Hirono et al. (2016) [71]	Japan	69 (41–90)	46	46:0	NCCN	CTx or concurrent CRTx
Jung et al. (2019) [74]	South Korea	64 (38–84)	95	0:95	NR	RTx, some with sequential CTx
Lee et al. (2015) [76]	South Korea	NEO + resection: 61.7 ± 8.77 NEO only: 59 ± 12.39	70 (42 analysable)	42:0	NCCN	Concurrent CRTx
Lee et al. (2024) [77]	South Korea	NR	662 (245 analysable)	147:98	NCCN	CTx
Leen et al. (2018) [78]	UK	63.4 (32–79)	75	0:75	NR	IRE, sequential with CTx
Liang et al. (2023) [80]	China	60 (39–71)	36	0:36	NCCN	CTx with or without sequential immunotherapy or RTx
Ma et al. (2023) [81]	China	NR	103	0:103	AJCC	Sequential CTx with IRE with or without immunotherapy
Matsumoto et al. (2023) [83]	Japan	68 (56–80)	39 (15 analysable)	0:15	NCCN	CTx, some with sequential RTx
Mellon et al. (2015) [84]	USA	66.5 (45–85)	159	110:49	NCCN	Sequential CTx and RTx
Mellon et al. (2016) [85]	USA	NEO: 65.9 (45–82)	220 (61 analysable)	56:5	NCCN	Sequential CTx and RTx
Murakami et al. (2017) [87]	Japan	NR	77 (52 analysable)	52:0	NCCN	CTx
Nerwal et al. (2025) [90]	Australia	65 (39–84)	37	32:5	NCCN	CTx, some with sequential CRTx
Okada et al. (2022) [91]	Japan	67 (60–72)	122	87:35	NCCN	CTx
Okamura et al. (2024) [92]	Japan	73 (54–86)	26	26:0	NCCN	Concurrent CRTx
Paik et al. (2015) [93]	South Korea	63 (36–85)	54	0:54	NCCN	Concurrent CRTx
Parsonson et al. (2021) [94]	Australia	66 (41–84)	48	48:0	NR	CTx, RTx or CRTx, both concurrent and sequential
Patel et al. (2014) [95]	USA	NEO + resection: 60 (39–72) NEO only: 65 (43–82)	39	39:0	NCCN	CTx or concurrent CRTx
Peng et al. (2019) [96]	USA	64.1 ± 11.0	71	71:0	NCCN	Concurrent CRTx, CTx, or CTx with sequential CRTx
Pietrasz et al. (2019) [98]	France	61.7 (28.3–78.9)	203	106:97	NCCN	CTx, some sequential with CRTx
Reddy et al. (2022) [99]	USA	73.6 (70.1–84.1)	57	27:30	NCCN	Sequential CTx and RTx
Seelen et al. (2023) [102]	USA, Netherlands	63.6 ± 9.6	168	0:168	NCCN	CTx or concurrent CRTx
Shrestha et al. (2017) [103]	USA	65 (42–83)	93	93:0	AHPBA/SSO/SSAT/NCCN	CTx, CRTx or CTx sequential with CRTx
Su et al. (2022) [106]	Taiwan	NR	117	117:0	International Association of Pancreatology	CTx
Sui et al. (2017) [108]	Japan	67.9 ± 8.5	93	0:93	NCCN and ISGPS	Concurrent CRTx
Templeton et al. (2021) [112]	Canada	65 (54–79)	20	20:0	NR	CTx
Truty et al. (2021) [115]	USA	64.1 (24.1–81.9)	194	123:71	ALLIANCE	Sequential CTx with CRTx
Tsujimoto et al. (2019) [116]	Japan	67 (47–75)	30	8:22	NCCN	CTx or sequential CTx and CRTx
Wijetunga et al. (2023) [24]	Australia	64.6 (33.1–79.1)	60	34:26	NCCN	CTx, some with sequential CRTx
Wolfe et al. (2020) [119]	USA	66 (32–86)	72	50:22	NCCN	CTx or concurrent CRTx, or CTx with sequential RTx
Yeung et al. (2024) [122]	UK	Group 1: 65.0 (57.5–72.5) Group 2: 70.0 (64.5–75.5)	54	21:33	NCCN	CTx, some sequential with CRTx
Yoo et al. (2019) [123]	South Korea	60 (30–78)	135	65:70	NCCN	CTx
Zakem et al. (2021) [124]	USA	66.2 (43.3–86)	103	85:18	NCCN	Sequential CTx and RTx
Case series						
Saito et al. (2017) [101]	Japan	71 (59–78)	7	0:7	NCCN	CTx

Abbreviations: AHPBA: The Americas Hepato-Pancreatico-Biliary Association, AJCC: American Joint Committee on Cancer, BRPC: borderline resectable pancreatic cancer, CRTx: chemoradiotherapy, CTx: chemotherapy, DPCG: Dutch Pancreatic Cancer Group, GSSC: Gastrointestinal Symposium Steering Committee, IRE: irreversible electroporation, ISGPS: International Study Group of Pancreatic Surgery, LAPC: locally advanced pancreatic cancer, MDACC: MD Anderson Cancer Center, NCCN: National Comprehensive Cancer Network, NR: not reported, RTx: radiotherapy, SSAT: Society for Surgery of the Alimentary Tract, SSO: Society of Surgical Oncology, UICC: Union for International Cancer Control.

**Table 2:** Grading of Recommendation, Assessment, Development and Evaluation (GRADE) assessment of outcomes summary table.

Outcomes	Effect Size (95% CI)	Number of Participants (Studies)	Certainty of Evidence	Importance
**Objective response rate**	0.27 (0.21–0.34)	1979 (29 studies)	Low ^a,b^	Critical
**Rate of proceeding to surgery**	0.47 (0.39–0.55)	4025 (43 studies)	Low ^a,b^	Critical
**Resection rate**	0.41 (0.34–0.48)	3723 (39 studies)	Low ^a,b^	Critical
**R0 status**	0.84 (0.79–0.89)	2378 (45 studies)	Low ^a,b^	Critical
**N0 status**	0.56 (0.49–0.62)	2044 (30 studies)	Low ^a,b^	Critical
**Overall survival**	0.36 (0.27–0.49)	828 (6 studies)	Moderate ^a^	Critical

^a^ Downgraded for risk of bias from included studies. ^b^ Downgraded for inconsistency due to study heterogeneity.

## Data Availability

The original contributions presented in this study are included in the article/Supplementary Material. Further inquiries can be directed to the corresponding author.

## Supplementary Materials

The following supporting information can be downloaded at https://www.mdpi.com/article/10.3390/cancers18071139/s1. Table S1: Detailed table of included studies, Table S2: Tumour responses of studies with heterogenous neoadjuvant regimens, Table S3: Rates of proceeding to surgery from studies with heterogenous neoadjuvant regimens, Table S4: Rates of resection and resection status from studies with heterogenous neoadjuvant regimens and resection, Table S5: Nodal status of study with heterogenous neoadjuvant regimens and resection, Table S6: Overall survival following neoadjuvant chemotherapy with or without resection, Table S7: Overall survival following neoadjuvant concurrent chemoradiotherapy with or without resection, Table S8: Overall survival following neoadjuvant sequential chemotherapy and chemoradiotherapy with or without resection, Table S9: Overall survival following neoadjuvant sequential chemotherapy and radiotherapy with or without resection, Table S10: Overall survival of studies with heterogenous neoadjuvant regimens with or without resection, Table S11: Recurrence-free survival or disease-free survival following neoadjuvant chemotherapy with or without resection, Table S12: Recurrence-free survival or disease-free survival following neoadjuvant chemoradiotherapy with or without resection, Table S13: Recurrence-free survival or disease-free survival following neoadjuvant sequential chemotherapy and chemoradiotherapy with or without resection, Table S14: Recurrence-free survival or disease-free survival from studies with heterogenous neoadjuvant regimens with or without resection, Table S15: Progression-free survival following neoadjuvant chemotherapy with or without resection, Table S16: Progression-free survival following neoadjuvant concurrent chemoradiotherapy with or without resection, Table S17: Progression-free survival following neoadjuvant sequential chemotherapy and radiotherapy with or without resection, Table S18: Progression-free survival of studies with heterogenous neoadjuvant regimens with or without resection, Table S19: Quality assessment of single-arm studies using Methodological Index for Non-Randomised Studies (MINORS) tool; Table S20: PRISMA 2020 Checklist. Figure S1: Risk-of-bias assessment of included articles using Version 2 of the Cochrane Risk-of-Bias Tool for Randomised Trials (RoB 2) [32,51,62,72,73,75,104,117], Figure S2: Risk-of-bias assessment of included articles using the Risk of Bias in Non-Randomised Studies of Interventions (ROBINS-I) tool [28,42,48,49,61,67,68,76,77,81,82,83,85,87,91,98,106,112,115,122,123,124], Figure S3: Assessment of small study effects with funnel plot and Egger’s test for objective response rate (*n* = 29). Plots representing individual studies, Figure S4: Assessment of small study effects with funnel plot and Egger’s test for rates of proceeding to surgery (*n* = 43). Plots representing individual studies, Figure S5: Assessment of small study effects with funnel plot and Egger’s test for rates of resection (*n* = 39). Plots representing individual studies, Figure S6: Assessment of small study effects with funnel plot and Egger’s test for resection status (*n* = 45). Plots representing individual studies, Figure S7: Assessment of small study effects with funnel plot and Egger’s test for nodal status (*n* = 30). Plots representing individual studies.

## Abbreviations

The following abbreviations are used in this manuscript:

AHPBA	The Americas Hepato-Pancreatico-Biliary Association
AJCC	American Joint Committee on Cancer
ASCO	American Society of Clinical Oncology
BRPC	Borderline resectable pancreatic cancer
CHA	Common hepatic artery
CI	Confidence interval
CR	Complete response
CRTx	Chemoradiotherapy
CTx	Chemotherapy
DFS	Disease-free survival
DPCG	Dutch Pancreatic Cancer Group
EBRT	External-beam radiation therapy
GRADE	Grading of Recommendation, Assessment, Development and Evaluation
GSSC	Gastrointestinal Symposium Steering Committee
HR	Hazard ratio
IMRT	Intensity-modulated radiation therapy
IRE	Irreversible electroporation
ISGPS	International Study Group of Pancreatic Surgery
LAPC	Locally advanced pancreatic cancer
MCW	Medical College of Wisconsin
MDACC	MD Anderson Cancer Center
MeSH	Medical subject heading
NCCN	National Comprehensive Cancer Network
NR	Not reported
ORR	Objective response rate
OS	Overall survival
PDAC	Pancreatic ductal adenocarcinoma
PFS	Progression-free survival
PR	Partial response
PRISMA	Preferred Reporting Items for Systematic Reviews and Meta-Analyses
PV	Portal vein
RFS	Recurrence-free survival
RoB 2	Version 2 of the Cochrane Risk-of-Bias Tool for Randomised Trials
ROBINS-I	Risk of Bias in Non-Randomised Studies of Interventions
RTx	Radiotherapy
SBRT	Stereotactic body radiation therapy
SMA	Superior mesenteric artery
SMV	Superior mesenteric vein
SSAT	Society for Surgery of the Alimentary Tract
SSO	Society of Surgical Oncology
UICC	Union for International Cancer Control

## Appendix A. Complete Search Strategy

**Table A1:** **Ovid MEDLINE (R) Epub Ahead of Print and In-Process, In-Data-Review and Other Non-Indexed Citations and Daily** <7 April 2025>.

	Searches	Results
1	“pancreatic cancer” OR “pancreas cancer” OR “pancrea * adj3 cancer” OR “Pancreatic ductal adenocarcinoma” OR “pancrea * adj3 adenocarcinoma”	647
2	“locally advanced pancreatic cancer” OR “LAPC” OR “borderline resectable pancreatic cancer” OR “resectable pancreatic cancer” OR “local adj3 pancrea * cancer”	10,145
3	(outcome * or survival * or disabilit * or mortality or morbidity).ti,kf.	1,208,698
4	exp morbidity/ or mortality/ or hospital mortality/ or survival rate/	99,625
5	(postoperative complication * or post-operative complication *).tw,kf.	113,508
6	“Survival” OR “Survival Outcomes” OR “Overall Survival” OR “Progression-Free Survival” OR “Disease-Free Survival”	4142
7	“Downstaging”	3377
8	1 and 2	2036
9	3 or 4 or 5 or 6 or 7	1,975,925
10	1 and 9	896
11	limit 10 to humans	811
12	limit 11 to english language	720

* was used as a truncation symbol used to broaden the search by finding multiple variations of the root word.

**Table A2:** **Ovid Embase Classic + Embase <1947 to 7 April 2025>**.

	Searches	Results
1	“pancreatic cancer” OR “pancreas cancer” OR “pancrea * adj3 cancer” OR “Pancreatic ductal adenocarcinoma” OR “pancrea * adj3 adenocarcinoma”	9354
2	“locally advanced pancreatic cancer” OR “LAPC” OR “borderline resectable pancreatic cancer” OR “resectable pancreatic cancer” OR “local adj3 pancrea * cancer”	122,292
3	(outcome * or survival * or disabilit * or mortality or morbidity).ti,kf.	1,660,660
4	exp morbidity/ or mortality/ or hospital mortality/ or survival rate/	1,633,229
5	(postoperative complication * or post-operative complication *).tw,kf.	16,328,312
6	“Survival” OR “Survival Outcomes” OR “Overall Survival” OR “Progression-Free Survival” OR “Disease-Free Survival”	16,222
7	“Downstaging”	6414
8	1 and 2	7071
9	3 or 4 or 5 or 6 or 7	2,950,605
10	1 and 9	1589
11	limit 10 to humans	1418
12	limit 11 to english language	1338

* was used as a truncation symbol used to broaden the search by finding multiple variations of the root word.

**Table A3:** **Cochrane Central Register of Controlled Trials <April 2025>**.

	Searches	Results
1	“pancreatic cancer” OR “pancreas cancer” OR “pancrea * adj3 cancer” OR “Pancreatic ductal adenocarcinoma” OR “pancrea * adj3 adenocarcinoma”	4421
2	“locally advanced pancreatic cancer” OR “LAPC” OR “borderline resectable pancreatic cancer” OR “resectable pancreatic cancer” OR “local adj3 pancrea * cancer”	10,017
3	(outcome * or survival * or disabilit * or mortality or morbidity).ti,kf.	96,538
4	exp morbidity/ or mortality/ or hospital mortality/ or survival rate/	35,759
5	(postoperative complication * or post-operative complication *).tw,kf.	15,998
6	“Survival” OR “Survival Outcomes” OR “Overall Survival” OR “Progression-Free Survival” OR “Disease-Free Survival”	11,624
7	“Downstaging”	554
8	1 and 2	214
9	3 or 4 or 5 or 6 or 7	149,910
10	1 and 9	818
11	limit 10 to humans	818
12	limit 11 to english language	782

* was used as a truncation symbol used to broaden the search by finding multiple variations of the root word.

**Table A4:** **Ovid Emcare <1995 to 2025 Week 15>**.

	Searches	Results
1	“pancreatic cancer” OR “pancreas cancer” OR “pancrea * adj3 cancer” OR “Pancreatic ductal adenocarcinoma” OR “pancrea * adj3 adenocarcinoma”	10,074
2	“locally advanced pancreatic cancer” OR “LAPC” OR “borderline resectable pancreatic cancer” OR “resectable pancreatic cancer” OR “local adj3 pancrea * cancer”	22,931
3	(outcome * or survival * or disabilit * or mortality or morbidity).ti,kf.	490,340
4	exp morbidity/ or mortality/ or hospital mortality/ or survival rate/	411,929
5	(postoperative complication * or post-operative complication *).tw,kf.	34,124
6	“Survival” OR “Survival Outcomes” OR “Overall Survival” OR “Progression-Free Survival” OR “Disease-Free Survival”	17,636
7	“Downstaging”	918
8	1 and 2	2653
9	3 or 4 or 5 or 6 or 7	809,444
10	1 and 9	2005
11	limit 10 to humans	1877
12	limit 11 to english language	1824

* was used as a truncation symbol used to broaden the search by finding multiple variations of the root word.
