# Supplementary material for: Systematic Review and Meta-Analysis of Current and Novel Approaches in the Management of Borderline Resectable and Locally Advanced Pancreatic Cancer
Source: Cancers (Basel). 2026 Apr 1;18(7):1139. doi: 10.3390/cancers18071139 (PMC13072174; doi:10.3390/cancers18071139)
Supplement: Supplementary file 1 [file cancers-18-01139-s001.zip › cancers-4214093-supplementary-proof.pdf]

Supplementary material

Table S1. Detailed table of included studies.

**Prospective studies**										
**Author (Year)**	Country of publication	Study design	Age (mean or median; range)	Total patients (n)	Sex (M:F)	BRPC : LAPC	Tumour location (n, %)	Reference definition	Treatment modality	Regimen (dose) and duration
Amodeo et al. (2018) [44]	USA	Phase I/II single arm clinical trial	64.5 (46-76)	24	15:9	13:11	Head/neck: 16 (66.7)<br>Body/tail: 7 (29.2)<br>Unrecorded: 1 (4.2)	NCCN	Concurrent CRTx	5-FU (200mg/m^2, daily)/oxaliplatin (30-60mg/m^2, weekly, dose escalation) and RTx (5040cGy, 25# followed by 540cGy, 3#) (n=24, n=20 completed all cycles). Total duration 5.5 weeks.<br><br>Those with stable disease had FOLFOX (oxaliplatin 85mg/m^2, leucovorin 350mg, 5-FU 400mg/m^2 bolus, 2,400mg/m^2 over 46hrs, biweekly) (n=14, n=9 completed all cycles). Total duration 12 week.
Belfiore et al. (2015) [46]	Italy	Prospective single arm study	69.2 (55-82)	20	10:10	0:20	Head/isthmus: 14 (70)<br>Body/tail: 6 (30)	NCCN	Sequential IRE and CTx	Nanoknife IRE, then gemcitabine (1000mg/m^2, biweekly)/oxaliplatin (100mg/m^2, biweekly) (n=20). Duration not reported.
Cascinu et al. (2021) [51]	Italy	Phase II randomised control trial	Group 1: 72 (42-79)<br>Group 2: 68 (36-74)	124	54:70	0:124	NR	NCCN	CTx	Gemcitabine (1000mg/m^2, weekly, 3x per 28-day cycle)/nab-paclitaxel (125mg/m^2, weekly, 3x per 28-day cycle) (n=63 completed all cycles). Total duration 84 days.<br><br>Gemcitabine alone (1000mg/m^2, weekly, 3x per 28-day cycle) (n=61, n=57 competed all cycles). Total duration 84 days.

										For those without resection, continued capecitabine (1250mg/m^2, daily on radiation days) and RTx (40-44.25 Gy, 15#) (n=40). Treatment duration not reported.
Chakraborty et al. (2014) [52]	USA	Phase II single arm clinical trial	66 (51-82)	13	5:8	13:0	NR	MDACC	Concurrent CRTx	Capecitabine (825mg/m^2, twice daily on radiation days, 40 doses total) with EBRT, either IMRT (n=11) or 3DCRT (n=2) (both 50Gy, 20#) (n=13). Total duration 4 weeks.
Du et al. (2023) [31]	China	Phase II single arm clinical trial	62 (40-75)	25	19:6	10:15	Head/uncinate: 13 (52)<br>Neck: 4 (16)<br>Body/tail: 8 (32)	NCCN	Concurrent immunotherapy with CRTx	Tislelizumab (200mg, three weekly)/nab-paclitaxel (125mg/m^2, weekly, 2x per 21-day cycle)/gemcitabine (1000mg/m^2 weekly, 2x per 21-day cycle) with SBRT (50Gy, 10#, then 30Gy, 10#) (n=25). Treatment duration 84 days.
Esnaola et al. (2014) [57]	USA	Phase II single arm clinical trial	60 (28-78)	37	20:17	13:24	Head: 29 (78.4)<br>Head/neck: 4 (10.8)<br>Head/neck/body: 1 (2.7)<br>Neck: 1 (2.7)<br>Neck/body: 1 (2.7)<br>Body: 1 (2.7)	NCCN	Concurrent biologic with CTx, some with sequential CRTx	Induction gemcitabine (1000 mg/m^2, biweekly)/oxaliplatin (100mg/m^2, biweekly) and cetuximab (400mg/m^2 initial dose, then 250mg/m^2, weekly) (n=37, n=34 completed all cycles). Treatment duration 84 days.<br><br>Those with stable disease had further capecitabine (800mg/m^2, twice daily on radiation days) with IMRT (54Gy, 30#) (n=26). Treatment duration not reported
Farnes et al. (2023) [42]	Norway	Prospective cohort study	69.5 (63-74)	230 (188 analysable)	98:90	96:92	Head/uncinate: 153 (81.4)<br>Body/tail: 35 (18.6)	NCCN	CTx	FOLFIRINOX (biweekly, median 4 cycles, range 3-5) (n=103), gemcitabine/nab-paclitaxel (weekly

									in a 21-day cycle, median 2 cycles, range 2-3.8) (n=48), gemcitabine alone (weekly in a 21-day cycle, median 2 cycles, range 1-3) (n=27), other (n=8). 30 patients required CTx switch. 2 patients had upfront resection and were excluded. Treatment duration and dose not reported.
Fietkau et al. (2021) [59]	Germany	Phase III randomised control trial	66 (41-79)	180	114:66	0:180	NR	NR	CTx, some with sequential CRTx	Induction gemcitabine (1000mg/m^2, weekly, 3x per 28-day cycle, n=43) or FOLFIRINOX (oxaliplatin 85mg/m^2, leucovorin 400mg/m^2, irinotecan 180mg/m^2, 5-FU 400mg/m^2 bolus then 2400mg/m^2 over 46hrs, biweekly, n=137). Treatment duration 71 days.<br><br>Followed by gemcitabine (300mg/m^2, weekly for 5 weeks; then 1000mg/m^2, weekly for 3 weeks) with RTx (either IMRT or 3DCRT) (50.4Gy, 28#) (n=62) or either gemcitabine alone (1000mg/m^2, weekly, 3x per 28-day cycle) or FOLFIRINOX (oxaliplatin 85mg/m^2, leucovorin 400mg/m^2, irinotecan 180mg/m^2, 5-FU 400mg/m^2 bolus then 2400mg/m^2 over 46hrs, biweekly) (n=64). Treatment duration 71 days.
Flak et al. (2019) [60]	Denmark	Phase I single arm clinical trial	67.1 (50-81)	33	18:15	0:33	Head: 20 (60.6)<br>Tail: 13 (39.4)	NCCN	IRE with or without	Nanoknife IRE (n=33), some with either prior FOLFIRINOX (n=18), gemcitabine combinations (n=9),

									sequential CTx or RTx	gemcitabine monotherapy (n=2) or other therapies (n=2), some with previous RTx alone (n=3). Treatment dose or duration not reported.
Ghaneh et al. (2023) [62]	UK, Germany	Phase II randomised control trial	63 (57-69)	86 (55 analysable)	26:29	55:0	NR	NCCN	CTx or concurrent CRTx	Gemcitabine (1000mg/m^2, weekly, 3x per 28-day cycle)/capecitabine (830mg/m^2, twice daily from days 1-21) (n=19). Treatment duration 56 days.<br><br>FOLFIRINOX (oxaliplatin 85mg/m^2, irinotecan 180mg/m^2, folinic acid given according to local practice, and 5-FU 400 mg/m^2 bolus, followed by 2400mg/m^2 over 46hrs, biweekly) (n=20). Treatment duration 56 days.<br><br>Capecitabine (830mg/m^2, twice daily on radiation days) and RTx (50.4Gy, 28#) (n=16). Treatment duration 5.5 weeks.
Goji et al. (2015) [63]	Japan	Phase I/II single arm clinical trial	68 (47-81)	25	11:14	0:25	Head: 11 (44)<br>Body/tail: 14 (56)	NR	Concurrent CRTx	Gemcitabine (300-400mg/m^2, on days 1, 8, 22, 29), S-1 (60mg/m^2, daily on days 1-5, 8-12, 22-26, 29-33) and RTx (50.4Gy, 28#) (n=25). Treatment duration 38 days.
Hammel et al. (2016) [65]	France, Australia, Belgium, Sweden	Phase III randomised control trial	63.3	442	232:110	0:442	Head: 302 (68.3)<br>Body/tail: 138 (31.2)<br>Unknown: 2 (0.5)	International Union Against Cancer staging system	CT or concurrent CRTx with or without concurrent biologic	Gemcitabine alone (1000mg/m^2, weekly, 3x per 28-day cycle) (n=223, n=135 completed all cycles) or gemcitabine (1000mg/m^2, weekly, 3x per 28-day cycle) with erlotinib (100mg, daily, increased to

										150mg in the maintenance phase) (n=219, n=134 completed all cycles). Treatment duration 112 days.<br><br>Followed by either the same chemotherapy regimen (n=136, unknown duration), or capecitabine (800mg/m^2, twice daily on radiation days, 60 doses total) and 3DCRT (54Gy, 30#) (n=133). Treatment duration 6 weeks.
Herman et al. (2015) [69]	USA	Phase II single arm clinical trial	67 (35-87)	49	31:18	0:49	Head: 41 (83.7)<br>Body/tail: 8 (16.3)	AHPBA/SSO/GSSC	RTx, some with sequential CTx	SBRT (33Gy, 5#) (n=49), some with previous gemcitabine (1000mg/m^2, weekly, median treatment duration 7 weeks, range 3-13, n=44). SBRT over a course of 1-2 weeks. Continued with gemcitabine (n=49, not treatment duration reported).
Hewitt et al. (2022) [70]	USA	Phase III randomised control trial	NR	303	153:150	53:250	NR	NCCN	CTx with or without sequential CRTx, some with cancer vaccine immunotherapy	FOLFIRINOX (oxaliplatin 85mg/m^2, irinotecan 180mg/m^2, leucovorin 400mg/m^2, 5-FU 2400mg/m^2 over 46hrs, biweekly, n=107) for 10 weeks or gemcitabine (1000mg/m^2, weekly, 3x per 28-day cycle)/nab-paclitaxel (125 mg/m^2, weekly, 3x per 28-day cycle) (n=51) for 84 days. For patients with stable/responding disease that was still unresectable, 5-FU (200-250mg/m^2, daily on radiation days) or capecitabine (825mg/m^2, twice daily on radiation days) and RTx (50.4Gy, 28#) were given.

										Treatment duration 5.5 weeks. N not reported<br><br>HAPa immunotherapy (300 million cells, weekly before CTx, biweekly after CTx) with FOLFIRINOX (oxaliplatin 85mg/m^2, irinotecan 180mg/m^2, leucovorin 400mg/m^2, 5-FU 2400mg/m^2 over 46hrs, biweekly) (n=100) or HAPa immunotherapy (300 million cells, weekly before CTx, biweekly after CTx) with gemcitabine (1000mg/m^2, weekly, 3x per 28-day cycle)/nab-paclitaxel (125 mg/m^2, weekly, 3x per 28-day cycle) (n=45). Treatment duration 17 weeks. For those with stable/responsive disease that was still unresectable, HAPa immunotherapy (300 millionm cells), 5-FU (200-250mg/m^2, daily on radiation days) or capecitabine (825mg/m^2, twice daily on radiation days) and RTx (50.4Gy, 28#) was given. Treatment duration 5.5 weeks. N not reported.
Ioka et al. (2021) [72]	Japan	Phase II randomised control trial	Arm A: 65 (38-78)<br>Arm B: 67 (28-80)	100	45:55	0:100	Head: 54 (54)<br>Body/tail: 46 (46)	UICC	CRTx with or without induction CTx	S-1 (80mg/m^2, twice daily on radiation days) with RTx (50.4Gy, 28#) for 5.5 weeks, followed by maintenance gemcitabine (1000mg/m^2, weekly, 3x per 28-day cycle) (n=44). Treatment duration not reported

										Gemcitabine (1000mg/m^2, weekly, 3x per 28-day cycle) for 16 weeks, followed by S-1 (80mg/m^2, twice daily on radiation days) with RTx (50.4Gy, 28#) for 5.5 weeks, then maintenance gemcitabine 1000mg/m^2, weekly, 3x per 28-day cycle) (n=32). Treatment duration not reported
Jang et al. (2018) [73]	South Korea	Phase II/III randomised control trial	59.4±8.4	27	17:10	27:0	Head: 23 (85.2)<br>Body/tail: 4 (14.8)	NCCN	Concurrent CRTx	Gemcitabine (400 mg/m^2, weekly), EBRT (3DCRT: 45Gy, 25# and 9Gy, 5#) (n=27, n=26 completed all cycles). Treatment duration 6 weeks.
Kunzmann et al. (2021) [75]	Germany	Phase II randomised control trial	Group 1: 61 (55-67)<br>Group 2: 63.5 (56-69)	130	66:64	0:130	Head: 95 (73.1)<br>Body/tail: 35 (26.9)	NCCN	CTx	Gemcitabine (1000mg/m^2, weekly, 3x per 28-day cycle)/nab-paclitaxel (125mg/m$^{2\cdot}$, weekly, 3x per 28-day cycle) (n=130). Treatment duration 56 days.<br><br>Followed by either gemcitabine (1000mg/m^2, weekly, 3x per 28-day cycle)/nab-paclitaxel (125mg/m$^{2\cdot}$, weekly, 3x per 28-day cycle) (n=64, n=60 completed all cycles), or FOLFIRINOX (oxaliplatin 85mg/m^2, leucovorin 400mg/m^2, irinotecan 180mg/m^2, and 5-FU 400mg/m^2 bolus followed by 2400mg/m^2 for 46hrs, biweekly) (n=66, n=52 completed all cycles). Treatment duration 8 weeks for both regimens.

Li et al. (2019) [79]	China	Phase II single arm clinical trial	62 (44-80)	41	22:19	0:41	Proximal: 24 (58.5) Distal: 17 (41.5)	NCCN	CTx	mFOLFIRINOX (oxaliplatin 68mg/m^2, leucovorin 400mg/m^2, irinotecan 135mg/m^2, 5-FU 2400mg/m^2 over 42hrs, biweekly) (n=41, n=35 received at least 4 cycles). Treatment duration at least 8 weeks.
Masui et al. (2016) [82]	Japan	Phase II non-randomsied clinical trial	63 (43-73)	18	8:10	18:0	Head: 13 (70.3) Body/tail: 5 (29.7)	NCCN	CTx	Gemcitabine (1000mg/m^2, weekly, 2x per 21-day cycle)/S-1 (80mg/m^2, twice daily for 14 days per 21-day cycle) (n=18). Treatment duration 63 days.
Moningi et al. (2015) [86]	USA	Single arm clinical trial	67.1 (35.6-87.5)	88	47:41	14:74	Head/uncinate/neck: 69 (78.4) Body/tail 19 (21.6)	AHPBA/SSO/SSAT	RTx, some with sequential CTx	SBRT (25-33Gy, 5#) (n=88), some with previous gemcitabine-based (n=59) or FOLFIRINOX-based (n=18) regimens. Treatment dose and duration not reported.
Murphy et al. (2019) [88]	USA	Phase II single arm clinical trial	63 (42-78)	49	23:26	0:49	Head: 31 (63.3) Body/tail: 19 (36.7)	NCCN	CTx, with sequential CRTx	FOLFIRINOX (oxaliplatin 85mg/m^2, leucovorin 400mg/m^2, irinotecan 180mg/m^2, and 5-FU 400mg/m^2 bolus followed by 2400mg/m^2 for 46hrs, biweekly) and losartan (daily, 25mg initial dose for 7 days, followed by 50mg) (n=49, n=39 completed all cycles). Treatment duration 16 weeks.<br><br>Patients downstaged with resectable disease and no vascular involvement received additional short course treatment of capecitabine (825mg/m^2, twice daily on radiation days) and RTx, either proton-based (25GyE, 5#) or photon-based (30Gy,

										10#) (n=7). Treatment duration 2 weeks.<br><br>Patients with vascular involvement had long course treatment of capecitabine (825mg/m^2, twice daily on radiotherapy days) or 5-FU (225mg/m^2, daily on radiation days) with IMRT (50.4Gy, 28#) (n=38). Treatment duration 5.5 weeks.
Nagakawa et al. (2017) [89]	Japan	Phase II single arm trial	65.1 (36-76)	27	21:6	27:0	Head: 11 (40.7)<br>Body/tail: 16 (59.3)	NCCN	Concurrent CRTx	Initial 2 doses of gemcitabine (1000mg/m^2). Gemcitabine (600mg/m^2, weekly)/S-1 (60mg, daily on days 1-14 and days 22-36) with IMRT (50.4Gy, 28#) (n=27, n=25 completed all cycles). Treatment duration 36 days
Philip et al. (2020) [97]	USA, France, Italy, Spain, Canada	Multicentrephase II single arm study	65.0 (60.0–72.0)	107	48:59	0:107	NR	AHPBA/SSO/SSAT	CTx	Gemcitabine (1000mg/m^2, weekly, 3x per 28-day cycle)/nab-paclitaxel (125mg/m^2, weekly, 3x per 28-day cycle) (n=107, n=62 completed all cycles). Treatment duration 168 days.<br><br>If not suitable for resection, received either gemcitabine/nab-paclitaxel (n=12) or CRTx with either capecitabine or gemcitabine (n=18). Treatment dose and duration according to institutional practice.
Picozzi et al. (2020) [32]	USA	Phase I/II randomised control trial	66	37	14:23	0:37	Head: 29 (78.4)<br>Body/tail: 12 (32.4)<br>Not mutually exclusive	NCCN	CTx, some concurrent with biologic	Pamrevlumab (35mg/kg, weekly, 2x per 28-day cycle, one additional dose in first cycle)/gemcitabine (1000mg/m^2, weekly, 3x per 28-day

										cycle)/nab-paclitaxel (125mg/m^2, weekly, 3x per 28-day cycle) (n=24, n=18 completed all cycles) or gemcitabine (1000mg/m^2, weekly, 3x per 28-day cycle)/nab-paclitaxel (125mg/m^2, weekly, 3x per 28-day cycle) (n=13, n=7 completed all cycles). Treatment duration 24-28 weeks.
Sahora et al. (2014) [100]	Austria	Phase II randomised control trial	62 (43-80)	32 (30 analysable)	12:18	11:19	Head: 24 (80)<br>Body/tail: 6 (20)	NCCN	Concurrent biologic with CTx	Gemcitabine (1000mg/m^2, weekly, 3x per 28-day cycle) with either a short (6-week, n=11) or long (12-week, n=19) course of bevacizumab (5mg/kg, biweekly). Treatment duration 16 weeks.
Snyder et al. (2024) [104]	USA	Phase II randomised control trial	63 (39-80)	126 (51 analysable)	21:30	51:0	NR	NCCN	CTx, some sequential with RTx	FOLFIRINOX (oxaliplatin 85mg/m^2, leucovorin 400mg/m^2, irinotecan 180mg/m^2, and 5-FU 2400mg/m^2 over 46hrs, biweekly) (n=70). Treatment duration 16 weeks.<br><br>FOLFIRINOX (oxaliplatin 85mg/m^2, leucovorin 400mg/m^2, irinotecan 180mg/m^2, and 5-FU 2400mg/m^2 over 46hrs, biweekly) with either SBRT (33-40Gy, 5#) or hypofractionated image-guide radiotherapy (25Gy, 5#) (n=56). Treatment duration 14 weeks.
Stein et al. (2016) [105]	USA	Phase II single arm trial	63 (46-79)	31	20:11	11:20	Head: 27 (87.1)<br>Body: 4 (12.9)	NCCN	CTx	mFOLFIRINOX (oxaliplatin 85mg/m^2, leucovorin 400mg/m^2, irinotecan 135mg/m^2, and 5-FU 300mg/m^2 bolus, then 2400mg/m^2

										over 46hrs, biweekly) (n=31). Treatment duration 16 weeks.
Sudo et al. (2017) [107]	Japan	Phase II single arm clinical trial	67.5 (44-79)	30	22:8	0:30	Head: 16 (53.3)<br>Body/tail: 14 (46.7)	NR	CTx, some with sequential CRTx, and further CTx	Gemcitabine (1000mg/m^2, weekly, 2x per 21-day cycle)/S-1 (either 60, 80 or 100mg/m^2, twice daily for 14 days per 21-day cycle) (n=30). Treatment duration 12 weeks.<br><br>Patients without distant metastasis then received S-1 (either 80, 100 or 120mg/m^2, daily on days 1-14 and days 22-35) and RTx (50.4Gy, 28#) (n=23). Treatment duration 5.5 weeks.<br><br>Maintenance S-1 (either 60, 80 or 100mg/m^2, daily for 14 days per 21-day cycle) following CRT (n=21). Treatment duration not reported.
Takahashi et al. (2016) [109]	Japan	Phase II single arm clinical trial	NR	184	112:72	184:0	Head: 149 (81)<br>Body: 35 (19)	NCCN	Concurrent CRTx	Gemcitabine (1000mg/m^2, weekly, 3x per 28-day cycle) and 3DCRT (50Gy, 20#) (n=184). Treatment duration 12 weeks.
Takahashi et al. (2022) [110]	Japan	Phase II single arm clinical trial	66 (48-75)	52	31:21	52:0	Head: 42 (80.8)<br>Body: 10 (19.2)	NCCN	Concurrent CRTx	S-1 (40mg/m^2, twice daily on radiation days) and RTx (50.4Gy, 28#) (n=52, n=50 completed all cycles). Treatment duration 5.5 weeks
Tasu et al. (2024) [111]	France	Phase II single arm trial	61 (37-77)	17	7:10	0:17	Head: 14 (82.4)<br>Body: 5 (29.4)<br>Uncinate: 6 (35.3)<br>Not mutually exclusive	NCCN	Sequential IRE with CTx	Gemcitabine/nab-paclitaxel or FOLFIRINOX for at least 3 months followed by IRE (n=17). Treatment doses not reported.

Temraz et al. (2022) [113]	Lebanon	Prospective single armstudy	61.8±7.9	49	31:18	20:29	Head/neck: 24 (49)<br>Body/tail: 25 (51)	NCCN	CTx	FOLFIRINOX (oxaliplatin 85mg/m^2, leucovorin 400mg/m^2, irinotecan 180mg/m^2, and 5-FU 400mg/m^2 bolus, then 2400mg/m^2 over 46hrs, biweekly) (n=49). Treatment duration median 11 cycles (range 2-31).
Teriaca et al. (2021) [114]	The Netherlands	Multicentre phase II single arm clinical trial	63 (40–74)	50	25:25	0:50	Head: 29 (58)<br>Body/tail: 21 (42)	DPCG	Sequential CTx and RTx	FOLFIRINOX (oxaliplatin 85mg/m^2, leucovorin 400mg/m^2, irinotecan 180mg/m^2, and 5-FU 400mg/m^2 bolus, then 2400mg/m^2 over 46hrs, biweekly) (n=50) followed by SBRT (40Gy, 5#) (n=39). Treatment duration median 8 cycles (range 2-8).
Versteijne et al. (2020) [117]	The Netherlands	Phase III randomised control trial	NR	54	NR	54:0	NR	UICC	Concurrent CRTx	Gemcitabine (1000mg/m^2, weekly, 3x per 28-day cycle, preceded and succeeded by 2x per 21-day cycle) with RTx (36Gy, 15#) (n=54). Treatment duration 10 weeks.
Weisz Ejlsmark et al. (2024) [118]	Denmark	Phase II single arm clinical trial	NR	28	12:16	0:28	NR	NCCN	RTx, some with sequential CTx	SBRT (50Gy, 5# for 7-8 days, n=26; or 60Gy, 8# for 10-13 days, n=2). Some patients had prior FOLFIRINOX (n=18), gemcitabine/nab-paclitaxel (n=5), gemcitabine/S-1 (n=2) or gemcitabine alone (n=2). Treatment duration and dose not reported.
Yabushita et al. (2023) [120]	Japan	Prospective single arm study	70 (37-89)	122	74:48	122:0	Head: 100 (82)<br>Body/tail: 22 (18)	NCCN	Concurrent CRTx	Gemcitabine (1000mg/m^2 on days 8 and 15)/S-1 (60mg/m^2 on days 1-14), treatment duration 30 days. Followed by S-1 (60mg/m^2, treatment duration not reported)

										with RTx (30Gy, 10# for 2 weeks) (n=122).
Yamaguchi et al. (2022) [121]	Japan	Phase II single arm clinical trial	Group 1: 66 (58-73)<br>Group 2: 66 (58-71)	51	29:22	51:0	Head: 38 (74.5)<br>Body/tail: 13 (25.5)	NCCN	CTx	FOLFIRINOX (oxaliplatin 85mg/m^2, leucovorin 200mg/m^2, irinotecan 180mg/m^2, and 5-FU 400mg/m^2 bolus, then 2400mg/m^2 over 46hrs, biweekly) (n=26). Treatment duration 8 weeks.<br><br>Gemcitabine (1000mg/m^2, weekly, 3x per 28-day cycle)/nab-paclitaxel (125mg/m^2, weekly, 3x per 28-day cycle) (n=25). Treatment duration 8 weeks.
					**Retrospective studies**					
**Author**	Country of publication	Study design	Age (mean or median; range)	Total patients (n)	Sex (M:F)	BRPC : LAPC	Tumour location (n, %)	Reference definition	Treatment modality	Regimen (dose) and duration
Banks et al. (2024) [28]	Australia	Retrospective cohort study of a prospective multicentre registry	NEO: <60 n=46, ≥60 n=82	152 (128 analysable)	66:62	128:0	NR	NR	CTx, CRTx or RTx	FOLFIRINOX (n=100), gemcitabine/nab-paclitaxel (n=28), gemcitabine alone (n=7), 5-FU-based regimen (n=6) CRTx (n=10), RTx (n=1). Treatment dose, regimens for CRTx and duration not reported.
Bednar et al. (2017) [45]	USA	Retrospective cohort study of a prospectively maintained registry	66.5 (36-86)	92	48:44	0:92	Head/neck: 64 (69.6)<br>Body/tail: 28 (30.4)	NCCN	CTx, some sequential with RTx	5-FU and/or gemcitabine-based regimen (n=31), FOLFIRINOX (n=21), nab-paclitaxel and gemcitabine (n=20), FOLFIRINOX and nab-paclitaxel/gemcitabine (n=20). Some with prior RTx (n=59). Treatment duration and doses not reported

Blazer et al. (2015) [47]	USA	Retrospective cohort study	62.4 (40-81)	43	23:20	18:25	Head: 25 (58.1)<br>Body/tail: 18 (41.9)	AHPBA/SSAT/SSO/GSSC	CTx or concurrent CRTx	Two patients prior had gemcitabine (300mg/m^2) and RTx for 5-6 weeks. Treatment duration not reported.<br><br>mFOLFIRINOX (oxaliplatin 85mg/m^2, irinotecan 165mg/m^2, and 5-FU 2400mg/m^2, biweekly) (n=43). Treatment duration mean 4.9 cycles, (range 1-14)<br><br>If the tumour was not resectable, gemcitabine (1000mg/m^2, weekly) with RTx (36Gy, 15#) (n=23) was given. Treatment duration 15 days<br><br>At the oncologist's discretion oxaliplatin (85mg/m^2, weekly, 2x per 28-day cycle), gemcitabine (1000mg/m^2, weekly, 3x per 28-day cycle) and RTx (30Gy, 15#) (n=3) was given instead. Treatment duration 56 days.
Botta et al. (2023) [48]	USA	Retrospective cohort study	70.6 (44-87)	52	21:31	33:19	Head: 38 (73.1)<br>Body/tail 14 (26.9)	NCCN	CTx, CRTx or sequential CTx and CRTx	CTx alone: Gemcitabine alone (n=1), gemcitabine/nab-paclitaxel (n=20) or FOLFIRINOX (n=5) for 3-6 cycles. Treatment duration and dose not reported.<br><br>CTx followed by CRTx: either gemcitabine/nab-paclitaxel (n=12) or FOLFIRINOX (n=1), followed by gemcitabine and RTx (25Gy, 5#) (n=13), or gemcitabine and RTx

									(25Gy, 5#) (n=13). Treatment duration and dose not reported.<br><br>Concurrent CRTx: gemcitabine alone and RTx (25Gy, 5#). Treatment duration and dose not reported.
Brada et al. (2021) [49]	The Netherlands	Post-hoc cohort analysis of a prospective multicentre registry	Resected: 62±9<br>Unresected: 62±9	418 (293 analysable)	142:151	0:293	Head: 189 (64.5)<br>Body/tail: 104 (35.5)	DPCG	CTx	FOLFIRINOX (4 or more cycles) (n=418). Treatment duration and dose not reported.
Brada et al. (2025) [50]	The Netherlands	Post-hoc cohort analysis of two prospective registries	66 (57-69)	103	49:54	0:103	Head: 76 (73.8)<br>Body/tail: 27 (26.2)	DPCG	CTx or concurrent CRTx	CTx: gemcitabine (n=2), mFOLFIRINOX (n=88), gemcitabine/nab-paclitaxel (n=3) or Concurrent CRTx: FOLFIRINOX and RTx (n=9). Treatment duration 4-8 cycles. Treatment dose not reported.
Chen et al. (2017) [53]	China	Retrospective cohort study	< 60 45 (45.9%)<br>>60 53 (54.1%)	98	52:46	0:98	Head: 70 (71.4)<br>Body/tail: 28 (28.6)	AJCC 6th or 7th edition	RTx	Not reported
Chen et al. (2022) [54]	Taiwan	Retrospective cohort study	63 (41-85)	57	35:22	0:57	Head: 39 (68.4)<br>Non head: 18 (31.6)	NR	RTx, some with sequential CTx, or concurrent CRTx	Induction CTx (n=36). RTx (n=57) at variable doses, including 55Gy, 25# (n=30), 50-50.4Gy, 25-28# (n=22), post-RTx chemotherapy (n=50). Of which some patients with concurrent CRTx (n=47) including gemcitabine-based (n=13), 5-FU/capcetiabine-based (n=18), cisplatin/oxaliplatin-based (n=4), S-1 based (n=10), other (n=2). Treatment duration, regimen and dose not reported.

Comito et al. (2023) [55]	Italy	Retrospective single arm study	71 (41-91)	142	NR	0:142	Head: 91 (64.1) Uncinate: 23 (16.2%) Body/tail: 23 (16.2%) Isthmus: 5 (3.5%)	AHPBA/SSO/SSAT	RTx, some with sequential CTx	SBRT (45Gy, 6#) alone (n=66) or some with CTx prior including gemcitabine alone (n=7), FOLFIRINOX (n=18), gemcitabine/nab-paclitaxel (n=17), gemcitabine/oxaliplatin (n=21), PEX-G (n=10) and others (n=3). Some with CTx after SBRT, including capecitabine-based (n=5), FOLFIRINOX (n=6), gemcitabine-based (n=22), irinotecan (n=2), or others (n=7). Treatment duration and dose not reported.
Dai et al. (2023) [56]	Canada	Retrospective cohort study	65.8±9.3	723	412:311	0:723	Head: 465 (64.3) Body/tail: 157 (21.7) Other: 101 (14)	NR	CTx, some with prior RTx or surgery	Either FOLFINRINOX (n=399) or gemcitabine/nab-paclitaxel (n=324). Some with prior RTx (n=25). Treatment duration and dose not reported.
Ferrone et al. (2015) [58]	USA	Retrospective cohort study	62 (38-77)	40	21:19	15:25	NR	AHPBA/SSO/SSAT	CTx or concurrent CRTx	FOLFIRINOX for 1-24 cycles (n=40). Some patients received additional 5-FU and RTx (n=14), 5-FU, RTx and intraoperative RTx (n=10), capecitabine and proton beam therapy (n=6), or other schemes (n=5). Treatment dose and duration not reported.
Fossaert et al. (2022) [61]	France	Retrospective cohort study	66	38	14:24	38:0	NR	MDACC	CTx	FOLFIRINOX (oxaliplatin 85mg/m^2, leucovorin 400mg/m^2, irinotecan 180mg/m^2, and 5-FU 2400mg/m^2, biweekly) (n=35), of which 3 switched to FOLFOX (oxaliplatin 85mg/m^2, leucovorin 400mg/m^2, and 5-FU 2400mg/m^2, biweekly)

										(n=3). Treatment duration not reported.<br><br>Gemcitabine (850mg/m^2, weekly, 2x per 21-day cycle) and oxaliplatin (100mg/m^2, weekly, 1x per 21-day cycle). Treatment duration not reported.
Gemenetzis et al. (2019) [23]	USA	Retrospective cohort study	65.2±10.1	415	195:220	0:415	Head: 174 (41.9)<br>Uncinate: 62 (14.9)<br>Neck: 51 (12.3)<br>Body/tail: 128 (30.4)	NCCN	CTx, some with sequential RTx	Gemcitabine/nab-paclitaxel (n=87), gemcitabine/capecitabine (n=11), both FOLFIRINOX and gemcitabine/nab-paclitaxel (n=72), mFOLFIRINOX (n=28), FOLFIRINOX (n=156), gemcitabine alone (n=19), 5-FU/capecitabine (n=3) or FOLFIRINOX/capecitabine (n=2). Some had RTx (n=111). 37 patients did not receive neoadjuvant CTx. Treatment duration and doses were not reported.
Groot et al. (2019) [64]	USA	Retrospective cohort study	62.7±9.4	231	128:103	138:93	NR	NCCN	CTx or concurrent CRTx	FOLFIRINOX (n=81), mFOLFIRINOX (n=17), FOLFIRINOX/gemcitabine (n=44), gemcitabine/nab-paclitaxel (n=28), gemcitabine/capecitabine (n=30), gemcitabine alone (n=31). Some of which had concurrent conventional RTx (n=81) or IMRT/SBRT (n=99). Specific duration and dose not reported.
Han et al. (2020) [66]	South Korea	Retrospective cohort study	62.1±10.8	26	18:8	26:0	NR	NCCN	CTx or concurrent CRTx or RTx	Capecitabine (1250mg/m^2, twice daily for 14 days every 3 weeks)

										(n=1). Treatment duration not reported<br><br>Gemcitabine (400mg/m^2, weekly, 6 weeks total) (n=7).<br><br>3DCRT (45Gy, 25#, 5 weeks; then a boost of 9Gy, 5#) (n=3).<br><br>FOLFIRINOX (oxaliplatin 85mg/m^2, leucovorin 400mg/m^2, irinotecan 180mg/m^2, and 5-FU 400mg/m^2 bolus, then 2400mg/m^2 over 46hrs, biweekly) (n=4). Treatment duration not reported<br><br>5-FU (500mg/m^2 on each of the first 3 days of radiation) and RTx (20Gy, 10#) (n=10). Treatment duration not reported<br><br>1 patient with unknown regimen.
He et al. (2018) [67]	USA	Retrospective cohort study	NR	186	105:81	87:99	NR	NR	Concurrent CRTx	FOLFIRINOX (n=83), gemcitabine-based (n=74) or single agent (n=29), with either EBRT (n=112) or SBRT (n=74). Treatment duration, regimens and doses not reported.
He et al. (2021) [68]	China	Retrospective cohort study	Group 1: 60 (39-80)<br>Group 2: 59 (39-70)<br>Group 3: 59 (34-87)	140	62:78	0:140	Head: 75 (53.6)<br>Tail: 65 (46.4)	NCCN	CTx with either conversion surgery or IRE	FOLFIRINOX (4-6 cycles) or gemcitabine/nab-paclitaxel (3 cycles) (n=140). Some with IRE (n=64). Treatment duration and dose not reported.

Hill et al. (2022) [43]	USA	Retrospective single arm study	66 (42-84)	155	80:75	91:64	Head/neck/uncinate: 108 (69.7)<br>Body/tail: 47 (30.3)	NCCN	CTx, with sequential RTx	FOLFIRINOX (n=116), gemcitabine/nab-paclitaxel (n=37), other (n=2). Treatment duration median 4 months (range 1-18). Followed by SBRT (median dose 33Gy, range 30-30; 5#).
Hirono et al. (2016) [71]	Japan	Retrospective cohort study	69 (41–90)	46	NR	46:0	Head: 14 (30.4)<br>Neck: 6 (13)<br>Tail: 20 (43.5)<br>Unrecorded: 6 (13)	NCCN	CTx or concurrent CRTx	Either S-1 (80mg/m^2, alternate day administration for 6 weeks) with EBRT (50gy, 25#) (n=26) or S-1 (80mg/m^2, alternate day administration for 9 weeks)/gemcitabine (800mg/m^2, on days 1, 8, 22, 29, 43, and 50) (n=20).
Jung et al. (2019) [74]	South Korea	Retrospective single arm study	64 (38-84)	95	49:46	0:95	Head: 52 (54.7)<br>Body/tail: 43 (45.3)	NR	RTx, some with sequential CTx	SBRT (median dose 28Gy, range 24-36Gy, 4#) (n=95) in 1 week. Some patients with prior induction CTx (n=13) and/or sequential CTx before or after SBRT (n=90). Specific regimen, treatment duration and dose not reported.
Lee et al. (2015) [76]	South Korea	Retrospective cohort study	NEO+resection: 61.7±8.77<br>NEO only: 59±12.39	70 (42 analysable)	19:23	42:0	NR	NCCN	Concurrent CRTx	Gemcitabine (1000mg/m^2, weekly for 5 weeks) and 3DCRT (either 45Gy, 50.4, or 58.4Gy, daily # at 1.8Gy). Some had additional cisplatin (70mg/m^2 on days 1 and 29) or capecitabine (40mg/m^2, daily on days 1-14 and days 21-35). Unknown patient breakdown, total n=42.
Lee et al. (2024) [77]	South Korea	Retrospective cohort study	NR	662 (245 analysable)	125:120	147:98	Head: 171 (69.8)<br>Body/tail: 72 (29.4)<br>Multicentric: 2 (0.8)	NCCN	CTx	FOLFIRINOX (n=202) or other regimen (n=43). Treatment duration and dose not reported.

Leen et al. (2018) [78]	UK	Retrospective single arm study	63.4 (32–79)	75	53:22	0:75	Head: 51 (68) Body/tail: 24 (32)	NR	IRE, sequential with CTx	FOLFIRINOX (n=28, of which 10 had dose reduction, 4 discontinued), gemcitabine/capecitabine (n=25), gemcitabine/platinum (n=12), or gemcitabine alone (n=10). Treatment duration 3-6 months. Followed by Nanoknife IRE (n=75). Treatment dose not reported.
Liang et al. (2023) [80]	China	Retrospective single arm study	60 (39-71)	36	18:18	0:36	NR	NCCN	CTx with or without sequential immunotherapy or RTx	mFOLFIRINOX (oxaliplatin 68mg/m^2, leucovorin 400mg/m^2, irinotecan 135mg/m^2, and 5-FU 2400mg/m^2, biweekly) (n=36), some with additional SBRT (25Gy, 5#) (n=15), some with additional anti PD-1 antibodies (no dose reported) (n=17). Treatment duration not reported
Ma et al. (2023) [81]	China	Retrospective cohort study	NR	103	31:72	0:103	Head: 79 (76.7) Body/tail: 24 (23.3)	AJCC	Sequential CTx with IRE with or without immunotherapy	Gemcitabine (1000mg/m^2) followed by IRE, with (n=25) or without (n=78) PD-1/PD-L1 blockade therapy, either camrelizumab (200 mg/2 weeks, n=10), toripalimab (240 mg/2 weeks, n=5), nivolumab (100 mg/2 weeks, n=4), pembrolizumab (100 mg/3 weeks, n=5), and atezolizumab (1200 mg/3 weeks, n=1). Treatment duration not reported.<br><br>Some patients had pre-experimental CTx, including gemcitabine (n=25), S-1 (n=36), FOLFIRINOX (n=44). Treatment duration and dose not reported.

Matsumoto et al. (2023) [83]	Japan	Retrospective cohort study	68 (56-80)	39 (15 analysable)	6:9	0:15	Head: 9 (60) Body/tail: 6 (40)	NCCN	CTx, some with sequential RTx	Gemcitabine alone (n=1), gemcitabine/nafamostat mesylate/S-1 (n=2), gemcitabine/nafamostat mesylate/S-1 later switched to gemcitabine/nab-paclitaxel (n=1), gemcitabine/nab-paclitaxel (n=7), gemcitabine/nab-paclitaxel and RTx (n=1), FOLFIRINOX (n=1), mFOLFIRINOX (n=1), S-1 (n=1). Treatment duration between 2-24 months. Treatment dose not reported.
Mellon et al. (2015) [84]	USA	Retrospective cohort study	66.5 (45-85)	159	87:72	110:49	Head: 131 (82.4) Tail: 28 (17.6)	NCCN	Sequential CTx and RTx	Either induction gemcitabine (750mg/m^2, weekly, 2x per 21-day cycle)/docetaxel (30mg/m^2, weekly, 2x per 21-day cycle)/capecitabine (750mg/m^2, twice daily on days 1-14 per 21-day cycle) (n=93) treatment duration 63 days; or induction FOLFIRINOX (oxaliplatin 85mg/m^2, leucovorin 400mg/m^2, irinotecan 180mg/m^2, and 5-FU 400mg/m^2 bolus, followed by 2400mg/m^2 over 46hrs, biweekly) (n=23) treatment duration 84 days; or induction gemcitabine alone (1000mg/m^2, weekly, 3x per 28-day cycle) (n=28) treatment duration 12 weeks; or induction gemcitabine (1000mg/m^2, weekly, 3x per 28-day cycle)/nab-paclitaxel (125mg/m^2, weekly, 3x per 28-day cycle) (n=8) or other regimens (n=6). Followed by SBRT (28-30Gy,

										5#) (n=93) treatment duration 5 days.
Mellon et al. (2016) [85]	USA	Retrospective cohort study	NEO: 65.9 (45–82)	220 (61 analysable)	36:25	56:5	Head: 54 (88.5)<br>Tail: 7 (11.5%)	NCCN	Sequential CTx and RTx	Gemcitabine (750mg/m^2, weekly, 2x per 21-day cycle)/docetaxel (30mg/m^2, weekly, 2x per 21-day cycle)/capecitabine (750mg/m^2, twice daily on days 1-14 per 21-day cycle) (n=48) treatment duration 63 days; or FOLFIRINOX (doses not reported, biweekly) (n=6) treatment duration 84 days; or gemcitabine/nab-paclitaxel (doses and treatment duration not reported) (n=1); or gemcitabine alone (doses and treatment duration not reported) (n=2); or other regimens (n=4). Followed by SBRT (30Gy, 5#)
Murakami et al. (2017) [87]	Japan	Retrospective cohort study	NR	77 (52 analysable)	35:17	52:0	Head: 35 (67.3)<br>Body/tail: 17 (32.7)	NCCN	CTx	Gemcitabine (1000mg/m^2, weekly, 2x per 21-day cycle)/S-1 (65mg/m^2, daily on days 1-14 per 21-day cycle) (n=52). Treatment duration 63 days.
Nerwal et al. (2025) [90]	Australia	Retrospective cohort study	65 (39-84)	37	16:21	32:5	Head: 32 (86.5)<br>Body/tail: 5 (13.5)	NCCN	CTx, some with sequential CRTx	FOLFIRINOX (n=32), gemcitabine/nab-paclitaxel (n=5). Some patients had further CRTx, either with capecitabine (n=14) or 5-FU (n=3) and RTx (54-50.4Gy, 1.8-2Gy fractions, 5-5.5 weeks). Treatment duration and doses not reported.
Okada et al. (2022) [91]	Japan	Retrospective cohort study	67 (60-72)	122	110:90	87:35	NR	NCCN	CTx	Gemcitabine (1000mg/m^2, weekly, 2x per 21-day cycle)/S-1 (65mg/m^2, daily on days 1-14 per 21-day cycle) treatment duration 63 days; or gemcitabine (either 800 or

										1000mg/m^2, biweekly)/nab-paclitaxel (either 100 or 125mg/m^2, biweekly)/S-1 (doses between 60-100mg/m^2, daily from days 1-7 of a 14-day cycle) treatment duration 84 days; or FOLFIRINOX (oxaliplatin 85mg/m^2, leucovorin 400mg/m^2, irinotecan 180mg/m^2, and 5-FU 400mg/m^2 bolus, then 2400mg/m^2 over 46hrs, biweekly) treatment duration 84 days. Patient breakdown not reported
Okamura et al. (2024) [92]	Japan	Retrospective single arm study	73 (54-86)	26	13:13	26:0	Head: 23 (88.5)<br>Body: 3 (11.5)	NCCN	Concurrent CRTx	S-1 (40mg/m^2, twice daily for 14 days per 21-day cycle) and IMRT (45Gy, 15#) (n=26). Treatment duration 6 weeks
Paik et al. (2015) [93]	South Korea	Retrospective single arm study	63 (36-85)	54	29:26	0:54	Head: 24 (44.4)<br>Body/tail: 30 (55.6)	NCCN	Concurrent CRTx	Either gemcitabine (400mg/m^2, weekly) (n=17), 5-FU (500mg/m^2, for 3 days at weeks 1 and 5) (n=9) or capecitabine (800mg/m^2, twice daily) (n=28) with EBRT (50.4Gy, 28#). Treatment duration and patient breakdown not reported.
Parsonson et al. (2021) [94]	Australia	Retrospective cohort study	66 (41-84)	48	25:23	48:0	Head: 38 (79.2)<br>Body/tail: 10 (20.8)	NR	CTx, RTx or CRTx, both concurrent and sequential	FOLFIRINOX (n=16), gemcitabine/nab-paclitaxel (n=13), gemcitabine (n=3), EBRT alone (n=2), EBRT with fluoropyrimidine (n=30), EBRT with gemcitabine (n=5), SBRT alone (n=5). Not mutually exclusive. Treatment duration and dose not reported.
Patel et al. (2014) [95]	USA	Retrospective cohort study	NEO+resection: 60 (39-72)	39	20:19	39:0	Head: 35 (89.7)<br>Neck: 2 (5.1)<br>Tail:2 (5.1)	NCCN	CTx or concurrent CRTx	Gemcitabine based (n=34), others (n=5). Some had RTx (200-

			NEO only: 65 (43-82)							5580cGy). Treatment duration, regimen and dose not reported.
Peng et al. (2019) [96]	USA	Retrospective cohort study	64.1±11.0	71	41:30	71:0	NR	NCCN	Concurrent CRTx, CTx, or CTx with sequential CRTx	Gemcitabine (n=28), 5-FU (n=4), capecitabine (n=22), other (n=2) with RTx.<br><br>Other regimens, including CTx alone (n=9), or CTx with sequential CRTx (n=6).<br><br>Treatment duration and dose not reported.
Pietrasz et al. (2019) [98]	France	Retrospective study of multicentric cohort study	61.7 (28.3–78.9)	203	119:84	106:97	Head/isthmus: 167 (82.2)<br>Body/tail: 36 (17.8)	NCCN	CTx, some sequential with CRTx	FOLFIRINOX (biweekly, median 6 cycles, range 1-30 cycles) alone (n=101), some with additional CRTx (n=102) involving either fluoropyrimidine-based therapy or gemcitabine, with either 3DCRT (n=20) or IMRT (n=82) (54Gy, 30#, 6 weeks). Treatment doses and duration were not reported.
Reddy et al. (2022) [99]	USA	Retrospective single arm study	73.6 (70.1-84.1)	57	31:26	27:30	Head: 33 (57.9)<br>Other: 24 (42.1)	NCCN	Sequential CTx and RTx	Either mFOLFIRINOX(n=30), gemcitabine/nab-paclitaxel (n=24), mFOLFIRINOX/capecitabine (n=1), gemcitabine/capecitabine (n=1), or gemcitabine (n=1). Then sequential SBRT (30-36Gy, 5#). Treatment duration and doses not reported.
Seelen et al. (2023) [102]	USA, Netherlands	Post-hoc cohort analysis of two prospective databases	63.6±9.6	168	76:92	0:168	NR	NCCN	CTx or concurrent CRTx	5-FU-based (n=92), gemcitabine-based (n=30), crossover (n=46). Treatment duration 3.4-7.2 months. Some of which had either concurrent conventional RTx (n=35)

										or SBRT (n=86). Treatment regimen and dose not reported.
Shrestha et al. (2017) [103]	USA	Retrospective cohort study	65 (42-83)	93	54:39	93:0	NR	AHPBA/SSO/SSAT/NCCN	CTx, CRTx or CTx sequential with CRTx	Gemcitabine-based (n=56), FOLFOX/FOLFIRINOX-based (n=13), both gemcitabine-based and FOLFIRINOX-based (n=3). 1 cycle 3-4 weeks, treatment duration median 3 cycles (range 1-9).<br><br>Either 5-FU, capecitabine or gemcitabine monotherapy with RTx (45-54Gy, 28-30#).<br><br>Some had CTx alone (n=14), CTx with sequential CRTx (n=58) or CRTx alone (n=19).
Su et al. (2022) [106]	Taiwan	Retrospective cohort study	NR	117	68:49	117:0	Head: 46 (39.3)<br>Body/tail: 53 (45.3)<br>Mixed: 18 (15.4%)	International Association of Pancreatology	CTx	Gemcitabine or S-1 monotherapy (n=9), S-1/leucovorin/oxaliplatin/gemcitabine (n=48), mFOLFIRINOX (n=21), gemcitabine/oxaliplatin/5-FU/leucovorin (n=21), others (n=18). Treatment duration and dose not reported.
Sui et al. (2017) [108]	Japan	Retrospective single arm study	67.9±8.5	93	55:38	0:93	Head: 57 (61.3)<br>Body/tail: 36 (38.7)	NCCN and ISGPS	Concurrent CRTx	Gemcitabine (800mg/m^2, weekly, 3x per 28-day cycle) (n=23) or S-1 (either 60, 80 or 100mg/m^2, twice daily for 14 days per 21-day cycle) (n=70) with IMRT (50Gy, 25#, 5.5 weeks) (n=93). Treatment duration not reported.
Templeton et al. (2021) [112]	Canada	Retrospective cohort study	65 (54-79)	20	14:6	20:0	Head: 17 (85)<br>Body: 3 (15)	NR	CTx	FOLFIRINOX (biweekly, median 6 cycles) (n=10) or gemcitabine/nab-paclitaxel (weekly, 3x per 28-day

										cycle, median 3 cycles) (n=10). Treatment dose not provided.
Truty et al. (2021) [115]	USA	Retrospective cohort study	64.1 (24.1-81.9)	194	107:87	123:71	Head/uncinate: 147 (75.8)<br>Body/tail: 47 (24.2)	ALLIANCE	Sequential CTx with CRTx	FOLFIRINOX (N=165) or gemcitabine (N=65) (standard dosing and cycling), variable treatment durations, of which 36 patients required switching. Median cycles given was 6, ranging from 2-21. Followed by CRTx with either capecitabine (n=134), 5-FU (n=45) or gemcitabine (n=15), with either proton or photon EBRT (50-50.4Gy, 25-28#, 5 weeks).
Tsujimoto et al. (2019) [116]	Japan	Retrospective single arm study	67 (47–75)	30	14:16	8:22	Head: 18 (60)<br>Tail: 12 (40)	NCCN	CTx or sequential CTx and CRTx	Gemcitabine/nab-paclitaxel (n=30) median treatment duration 5.4 months, ranging from 1.4-25.8 months. Some with sequential CRTx with S-1 (n=12) and maintenance therapy of either gemcitabine/nab-paclitaxel (n=4), S-1 (n=5), or gemcitabine/S-1 (n=1). Treatment duration and doses not reported.
Wijetunga et al. (2023) [24]	Australia	Retrospective cohort study of a prospectively maintained databse	64.6 (33.1–79.1)	60	25:35	34:26	NR	NCCN	CTx, some with sequential CRTx	FOLFIRINOX (n=21), gemcitabine/nab-paclitaxel (n=31), gemcitabine/oxaliplatin (n=1), gemcitabine alone (n=5), oxaliplatin alone (n=1), capecitabine alone (n=1). Median treatment duration 4 cycles (range 2-13). Some with sequential capecitabine and IMRT (45Gy, 25#) treatment duration 5-6 weeks (n=29). Treatment doses not reported.

Wolfe et al. (2020) [119]	USA	Retrospective cohort study	66 (32-86)	72	29:43	50:22	Head/neck: 48 (66.7) Body/tail: 24 (33.3)	NCCN	CTx or concurrent CRTx, or CTx with sequential RTx	mFOLFIRINOX (oxaliplatin, 85mg/m^2, irinotecan 165-180mg/m^2, leucovorin followed by 5-FU 2400 mg/m^2 over 46hrs, biweekly) (n=52); or gemcitabine (1000mg/m^2, biweekly)/nab-paclitaxel (125mg/m^2, weekly, 2x per 28-day cycle, unknown treatment duration) (n=20). For those deemed unresectable or high risk of positive margins, gemcitabine (1000mg/m^2, weekly) (n=37) or 5-FU/capecitabine (no dose given) (n=5) was given with 3DCRT or IMRT. Median dose 36Gy (30-54). 3 patients had RTx alone. Treatment doses and duration not reported.
Yeung et al. (2024) [122]	UK	Retrospective cohort study	Group 1: 65.0 (57.5-72.5) Group 2: 70.0 (64.5-75.5)	54	30:24	21:33	NR	NCCN	CTx, some sequential with CRTx	FOLFIRINOX, gemcitabine/capecitabine or other (n=54) some with further capecitabine and RTx (36-54Gy, 15-30#) (n=37). Treatment doses and duration not reported.
Yoo et al. (2019) [123]	South Korea	Retrospective cohort study	60 (30-78)	135	72:63	65:70	NR	NCCN	CTx	Gemcitabine-based (n=69) or FOLFIRINOX (n=66). Treatment doses and duration not reported.
Zakem et al. (2021) [124]	USA	Retrospective cohort study	66.2 (43.3–86)	103	52:51	85:18	Head: 87 (84.5) Body: 16 (15.5)	NCCN	Sequential CTx and RTx	FOLFIRINOX (n=69), gemcitabine/nab-paclitaxel (n=28), or others (n=6) (2-3 months), then SBRT (30-33Gy) (n=103). Treatment duration and dose not reported.
**Case series**										

**Author**	Country of publication	Study design	Age (mean or median; range)	Total patients (n)	Sex (M:F)	BRPC : LAPC	Tumour location (n, %)	Reference definition	Treatment modality	Regimen (dose)
Saito et al. (2017) [101]	Japan	Single arm case series	71 (59–78)	7	1:6	0:7	Head: 6 (85.7) Tail: 1 (14.3)	NCCN	CTx	Gemcitabine (1000mg/m^2, weekly, 3x per 28-day cycle) and nab-paclitaxel (125mg/m^2, weekly, 3x per 28-day cycle). Median 4 cycles (2-7) (n=7)

Abbreviations: AHPBA: The Americas Hepato-Pancreatico-Biliary Association, AJCC: American Joint Committee on Cancer, BRPC: Borderline resectable pancreatic cancer, CRTx: chemoradiotherapy, CTx: chemotherapy, DPCG: Dutch Pancreatic Cancer Group, EBRT: External beam radiation therapy, GSSC: Gastrointestinal Symposium Steering Committee, IMRT: Intensity modulated radiation therapy, IRE: Irreversible electroporation, ISGPS: International Study Group of Pancreatic Surgery, LAPC: Locally advanced pancreatic cancer, MDACC: MD Anderson Cancer Center, NCCN: National Comprehensive Cancer Network, NR: not reported, RTx: radiotherapy, SBRT: stereotactic body radiation therapy, SSAT: Society for Surgery of the Alimentary Tract, SSO: Society of Surgical Oncology, UICC: Union for International Cancer Control.

Table S2: Tumour responses of studies with heterogenous neoadjuvant regimens

Abbreviations: CRTx: chemoradiotherapy, CTx: chemotherapy, NR: not reported RTx: radiotherapy

**Author**	**Year**	**Complete response (n, % total)**	**Near complete response (n, % total)**	**Partial response (n, % total)**	**Stable disease (n, % total)**	**Poor, limited or no response (n, % total)**	**Progressive disease (n, % total)**	**Total patients (n)**	**Scoring system**	**Treatment duration**	**Treatment**
Blazer	2014	-	-	9 (22.5)	25 (62.5)	-	6 (15)	43 (40 analysed)	NR	Variable duration	CTx or concurrent CRTx
Chen	2022	1 (1.75)	-	4 (7.02)	45 (78.95)	-	7 (12.28)	57	RECIST	NR	RTx, some with sequential CTx or concurrent CRTx
Comito	2023	1 (0.7)	-	31 (21.83)	81 (57.04)	-	29 (20.42)	142	RECIST	NR	RTx, some with sequential CTx
Ferrone	2015	6 (15)	-	30 (75)	4 (10)	-	-	40	NR	Variable duration	CTx or concurrent CRTx
Gemenetzis	2019	2 (1.72)	-	11 (9.48)	96 (82.76)	-	7 (6.03)	415 (116 analysable)	RECIST	NR	CTx, some with sequential RTx
Hirono	2016	-	-	6 (13.04)	34 (73.91)	-	6 (13.04)	46	RECIST	Variable duration	CTx or concurrent CRTx
Liang	2023	-	-	9 (30)	17 (56.67)	-	2 (6.67)	36 (30 analysable)	NR	NR	CTx with or without sequential PD-1 inhibitor or RTx

Parsonson	2021	-	-	21 (43.75)	15 (31.25)	-	12 (25)	48	RECIST	NR	CTx, RTx or CRTx, both concurrent and sequential
Peng	2019	4 (5.63)	12 (16.90)	42 (59.15)	-	13 (18.31)	-	71	College of American Pathologists	NR	Concurrent CRTx, CTx, or CTx with sequential CRTx
Sudo	2017	-	-	10 (34.48)	19 (65.52)	-	-	30 (29 analysable)	RECIST	Variable duration	CTx, some with sequential CRTx, and further CTx
Wolfe	2020	1 (1.39)	-	17 (23.61)	49 (68.06)	-	5 (6.94)	72	RECIST	Variable duration	CTx or concurrent CRTx, or CTx with sequential RTx

Table S3: Rates of proceeding to surgery from studies with heterogenous neoadjuvant regimens

Abbreviations: CRTx: chemoradiotherapy, CTx: chemotherapy, IRE: irreversible electroporation, NR: not reported RTx: radiotherapy

**Author**	**Year**	**Progressed to surgery (n)**	**Did not progress to surgery (n)**	**Total patients (n)**	**Percentage progressed to surgery (%)**	**Treatment duration**	**Treatment**
Bednar	2017	19	73	92	(20.65)	NR	CTx, some with sequential RTx
Blazer	2014	31	12	43	(72.09)	Variable duration	CTx or concurrent CRTx
Brada	2025	103	0	103	(100%)	Variable duration	CTx or concurrent CRTx
Comito	2023	4	138	142	(2.82)	NR	RTx, some with sequential CTx
Dai	2023	89	634	723	(12.31)	NR	CTx, some with prior RTx or surgery
Ferrone	2015	40	0	40	(100)	Variable duration	CTx or concurrent CRTx
Fietkau	2021	36	144	180	(20)	Variable duration	CTx, some with sequential CRTx
Flak	2019	3	30	33	(9.09)	NR	IRE with or without sequential CTx or RTx
Gemenetzis	2019	116	262	415 (378 analysable)	(30.69)	NR	CTx, some with sequential RTx
Groot	2019	231	0	231	(100)	NR	CTx or concurrent CRTx
Hammel	2016	18	424	442	(4.07)	Variable duration	CT or concurrent CRTx with or without concurrent erlotinib

Han	2020	10	16	26	(38.46)	NR	CTx or concurrent CRTx or RTx
Herman	2015	4	45	49	(8.16)	Variable duration	RTx, some with sequential CTx
Hewitt	2022	41	117	158	(25.95)	Variable duration	CTx, some with sequential CRTx
Hirono	2016	40	6	46	(86.96)	Variable duration	CTx or concurrent CRTx
Jung	2019	7	88	95	(7.37)	NR	RTx, some with sequential CTx
Liang	2023	36	0	36	(100)	NR	CTx with or without sequential PD-1 inhibitor or RTx
Moningi	2015	19	69	88	(21.59)	NR	RTx, some with sequential CTx
Nerwal	2025	23	13	37 (36 analysable)	(63.89)	NR	CTx, some with sequential CRTx
Parsonson	2021	24	24	48	(50)	NR	CTx, RTx or CRTx, both concurrent and sequential
Patel	2014	17	0	17	(100)	NR	CTx or concurrent CRTx
Peng	2019	71	0	71	(100)	NR	Concurrent CRTx, CTx, or CTx with sequential CRTx
Seelen	2023	168	0	168	(100)	NR	CTx or concurrent CRTx
Shrestha	2017	48	43	93 (91 analysable)	(52.75)	Variable duration	CTx, CRTx or CTx sequential with CRTx
Sudo	2017	5	25	30	(16.67)	Variable duration	CTx, some with sequential CRTx, and further CTx
Weisz Ejlsmark	2024	6	22	28	(21.43)	NR	RTx, some with sequential CTx
Wijetunga	2021	60	0	60	(100)	Variable duration	CTx, some with sequential CRTx
Wolfe	2020	72	0	72	(100)	Variable duration	CTx or concurrent CRTx, or CTx with sequential RTx

Table S4: Rates of resection and resection status from studies with heterogenous neoadjuvant regimens and resection

Abbreviations: CRTx: chemoradiotherapy, CTx: chemotherapy, IRE: irreversible electroporation, RTx: radiotherapy

**Author**	**Year**	**R0 resection (n, % total resected)**	**R1 resection (n, % total resected)**	**R2 resection (n, % total resected)**	**Unspecified margins (n, % total resected)**	**Total resected (n, % total)**	**Explored with no resection (n, % total)**	**Decision not to resect or undergo surgery (n, % total)**	**Total patients (n)**	**Treatment duration**	**Treatment**

Bednar	2017	14 (73.68)	-	-	5 (26.32)	19 (20.65)	-	73 (79.35)	92	NR	CTx, some with sequential RTx
Blazer	2014	19 (86.36)	-	-	3 (13.64)	22 (51.16)	9 (20.93)	12 (27.91)	43	Variable duration	CTx or concurrent CRTx
Brada	2025	55 (53.4)	-	-	48 (46.6)	103 (100)	-	-	103	Variable duration	CTx or concurrent CRTx
Comito	2023	4 (100)	-	-	-	4 (2.82)	-	138 (97.18)	142	NR	RTx, some with sequential CTx
Dai	2023	50 (84.75)	-	-	9 (15.25)	59 (8.51)	-	634 (91.49)	723 (693 analysable)	NR	CTx, some with prior RTx and surgery
Ferrone	2015	35 (92)	-	-	5 (8)	40 (100)	-	-	40	Variable duration	CTx or concurrent CRTx
Fietkau	2021	25 (69.44)	-	-	11 (30.56)	36 (20)	-	144 (80)	180	Variable duration	CTx, some with sequential CRTx
Flak	2019	2 (66.67)	-	-	1 (33.33)	3 (9.09)	-	30 (90.91)	33	NR	IRE with or without sequential CTx or RTx
Gemenetzis	2019	75 (89.29)	9 (2.17)	-	-	84 (22.22)	32 (8.47)	262 (69.31)	415 (378 analysable)	NR	CTx, some with sequential RTx
Groot	2019	180 (77.92)	-	-	51 (22.08)	231 (100)	-	-	231	NR	CTx or concurrent CRTx
Hammel	2016	11 (61.11)	2 (11.11)	-	5 (2.78)	18 (4.26)	-	424 (95.93)	442	Variable duration	CT or concurrent CRTx with or without concurrent erlotinib
Han	2020	7 (70)	3 (30)	-	-	10 (38.46)	-	16 (61.54)	26	NR	CTx or concurrent CRTx or RTx
Herman	2015	4 (100)	-	-	-	4 (8.16)	-	45 (91.84)	49	Variable duration	RTx, some with sequential CTx
Hewitt	2022	-	-	-	41 (100)	41 (25.95)	-	117 (74.05)	158	Variable duration	CTx, some with sequential CRTx
Hirono	2016	32 (80)	8 (20)	-	-	40 (86.96)		6 (13.04)	46	Variable duration	CTx or concurrent CRTx
Jung	2019	4 (57.14)	-	-	3 (42.86)	7 (7.37)	-	88 (92.63)	95	NR	RTx, some with sequential CTx
Liang	2023	34 (94.44)	2 (5.56)	-	-	36 (100)	-	-	36	NR	CTx with or without sequential PD-1 inhibitor or RTx
Moningi	2015	16 (84.21)	-	-	3 (15.79)	19 (21.59)	-	69 (78.41)	88	NR	RTx, some with sequential CTx
Nerwal	2025	14 (70)	6 (30)	-	-	20 (54.05)	3 (8.11)	13 (35.14)	37	NR	CTx, some with sequential CRTx

Parsonson	2021	18 (75)	6 (25)	-	-	24 (50)	-	24 (50)	48	NR	CTx, RTx or CRTx both concurrent and sequential
Patel	2014	12 (70.59)	-	-	5 (29.41)	17 (100)	-	-	17	NR	CTx or concurrent CRTx
Peng	2019	51 (71.83)	20 (28.17)	-	-	71 (100)	-	-	71	NR	Concurrent CRTx, CTx, or CTx with sequential CRTx
Seelen	2023	128 (76.19)	40 (23.81)	-	-	168 (100)	-	-	168	NR	CTx or concurrent CRTx
Shrestha	2017	26 (68.42)	2 (5.26)	-	10 (26.32)	38 (40.86)	10 (10.75)	45 (48.39)	93	Variable duration	CTx, CRTx or CTx sequential with CRTx
Sudo	2017	3 (60)	-	-	2 (40)	5 (16.67)	-	25 (83.33)	30	Variable duration	CTx, some with sequential CRTx, and further CTx
Weisz Ejlsmark	2024	4 (66.67)	2 (33.33)	-	-	6 (21.43)	-	22 (78.57)	28	NR	RTx, some with sequential CTx
Wijetunga	2021	32 (53.33)	28 (46.67)	-	-	60 (100)	-	-	60	Variable duration	CTx, some with sequential CRTx
Wolfe	2020	53 (73.61)	-	-	19 (23.39)	72 (100)	-	-	72	Variable duration	CTx or concurrent CRTx, or CTx with sequential RTx

Table S5: Nodal status of study with heterogenous neoadjuvant regimens and resection

Abbreviations: CRTx: chemoradiotherapy, CTx: chemotherapy, RTx: radiotherapy

**Author**	**Year**	**N0 (n, % total)**	**N1 (n, % total)**	**N2 (n, % total)**	**Positive nodes – unspecified (n, % total)**	**Total patients (n)**	**Treatment duration**	**Treatment**
Dai	2023	28 (47.6)	25 (42.37)	6 (10.17)	-	89 (59 analysable)	NR	CTx, some with prior RTx and surgery
Ferrone	2015	26 (65)	-	-	14 (35)	40	Variable duration	CTx or concurrent CRTx
Gemenetzis	2019	63 (75)	-	-	21 (25)	84	NR	CTx, some with sequential RTx
Groot	2019	140 (60.6)	72 (31.17)	19 (8.23)	-	231	NR	CTx or concurrent CRTx
Han	2020	6 (60)	-	-	4 (40)	10	NR	CTx or concurrent CRTx or RTx
Herman	2015	4 (100)	-	-	-	4	Variable duration	RTx, some with sequential CTx
Hirono	2016	9 (22.5)	-	-	31 (77.5)	40	Variable duration	CTx or concurrent CRTx

Liang	2023	11 (30.56)	14 (38.89)	11 (30.56)	-	36	NR	CTx with or without sequential PD-1 inhibitor or RTx
Matsumoto	2023	9 (60)	-	-	6 (40)	15	Variable duration	CTx, or CTx with sequential RTx
Moningi	2015	14 (73.68)	-	-	5 (2.63)	19	NR	RTx, some with sequential CTx
Parsonson	2021	10 (41.67)	14 (58.33)	-	-	24	NR	CTx, RTx or CRTx both concurrent and sequential
Patel	2014	10 (66.67)	5 (33.33)	-	-	17 (15 analysable)	NR	CTx or concurrent CRTx
Peng	2019	39 (54.93)	32 (45.07)	-	-	71	NR	Concurrent CRTx, CTx, or CTx with sequential CRTx
Seelen	2023	108 (64.29)	-	-	60 (35.71)	168	NR	CTx or concurrent CRTx
Wijetunga	2021	25 (41.67)	-	-	35 (58.33)	60	Variable duration	CTx, some with sequential CRTx
Wolfe	2020	32 (44.44)	-	-	40 (55.56)	72	Variable duration	CTx or concurrent CRTx, or CTx with sequential RTx

Table S6: Overall survival following neoadjuvant chemotherapy with or without resection

Abbreviations: CTx: chemotherapy, NR: not reported, OS: overall survival

**Author**	**Year**	**OS – Resected (months)**	**n**	**OS – Unresected (months)**	**n**	**P-value**	**HR**	**CI**	**Total patients (n)**	**Treatment duration**	**Treatment**
Brada	2021	24 (95% CI 19–38)	89	15 (95% CI 15–17)	204	<0.01	0.416	0.288–0.602	418 (293 analysable)	Variable duration	CTx - FOLFIRINOX
Fossaert	2022	26.6	38	-	-	-	-	-	38	NR	CTx – various regimens
He	2021	25.3 (95% CI, 15.9–33.3)	45	8.7 (95% CI, 4.4–22.0)	31	<0.001	0.274	0.133–0.564	140 (76 analysable)	NR	CTx – various regimens
Kunzmann	2021	27.5	52	13.9	78	<0·0001	0.34	0.21-0.54	130	16 weeks	CTx - gemcitabine/nab-paclitaxel with either FOLFIRINOX or gemcitabine/nab-paclitaxel
Lee	2024	43.2 (95%CI 38.4-48.1)	98	21.5 (95% CI, 19.7 to 23.3)	211	<0·001	-	-	662 (309 analysable)	NR	CTx – various regimens
Li	2019	27.7	12	13.2	27	NR	-	-	41 (39 analysable)	Variable duration	CTx - mFOLFIRINOX
Masui	2016	21.7	15	-	-	-	-	-	18 (15 analysable)	9 weeks	CTx - gemcitabine/S-1
Murakami	2017	27.2	47	-	-	-	-	-	52 (47 analysable)	9 weeks	CTx - gemcitabine/S-1
Okada	2022	29	122			-	-	-	122	Variable duration	CTx – various regimens

Pietrasz	2019	35.5 (95% CI 30.4–40.6)	101	-	-	-	-	-	101	Variable duration	CTx - FOLFIRINOX
Su	2022	29.1 (95% CI, 26.4-not reached)	34	14.3 (95% CI, 13.4-18.3)	83	<0.0001	0.2	0.10-0.40	117	NR	CTx – various regimens
Templeton	2021	32	5	16 (95% CI, 7.5–24.5)	15	0.016	-	-	20	Variable duration	CTx – various regimens
Yeung	2024	R0: 42	13	-	-	-	-	-	17	NR	CTx – various regimens
		R1: 7.5	4								
Yoo	2019	29.7 (95% CI, 22.5–36.8)	135	-	-	-	-	-	135	NR	CTx – various regimens

Table S7: Overall survival following neoadjuvant concurrent chemoradiotherapy with or without resection

Abbreviations: CRTx: chemoradiotherapy, NR: not reported, OS: overall survival

Author	Year	OS – Resected (months)	n	OS - Unresected (months)	n	P value	HR	CI	Total patients (n)	Treatment duration	Treatment
Amodeo	2018	31.65	2	10.4	18	NR	-	-	20	5.5 weeks	Concurrent CRTx with 5-FU and oxaliplatin
Lee	2015	30.9 (+/- 21.46)	30	19.5 (+/- 10.41)	12	0.006	-	-	42	NR	Concurrent CRTx (gemcitabine based)
Nagakawa	2017	22.9	19	9.3	8	0.025	-	-	27	36 days	Concurrent CRTx with gemcitabine and S-1
Okamura	2024	28	26	-	-	-	-	-	26	6 weeks	Concurrent CRTx with S-1
Takahashi	2022	41.8 (95% CI 27.0–56.6)	29	18.4	23	NR	-	-	52	5.5 weeks	Concurrent CRTx with S-1

Table S8: Overall survival following neoadjuvant sequential chemotherapy and chemoradiotherapy with or without resection

Abbreviations: CRTx: chemoradiotherapy, CTx: chemotherapy, NR: not reported, OS: overall survival

Author	Year	OS – Resected (months)	n	OS - Unresected (months)	n	P Value	HR	CI	Total patients (n)	Treatment duration	Tx modality
Murphy	2019	33 (95% CI, 31.4 to not reached)	34	-	-	-	-	-	34	Variable duration	Sequential CTx (FOLFIRINOX and losartan)

											and CRTx (with either capecitabine or 5-FU)
Pietrasz	2019	57.8 (95% CI 42.6–73.0)	102	-	-	-	-	-	102	Variable duration	Sequential CTx (FOLFIRINOX) and CRTx (various regimens)
Truty	2021	58.8	194	-	-	-	-	-	194	Variable duration	Sequential CTx and CRTx – various regimens
Yabushita	2023	24.7	94	9.6	28	<0.001	-	-	122	~6 weeks	Sequential CTx (gemcitabine/S-1) and CRTx with S-1
Yeung	2023	R0: 51<br>R1: 23	23<br>14	-	-	-	-	-	37	NR	Sequential CTx (various regimens) and CRTx (with capecitabine)

Table S9: Overall survival following neoadjuvant sequential chemotherapy and radiotherapy with or without resection

Abbreviations: CTx: chemotherapy, NR: not reported, OS: overall survival, RTx: radiotherapy, SBRT: stereotactic body radiatiotherapy

**Author**	**Year**	**OS – Resected (months)**	**n**	**OS - Unresected (months)**	**n**	**P-value**	**HR**	**CI**	**Total patients (n)**	**Treatment duration**	**Treatment**
Hill	2022	27 (95% CI 20.9-NR)	107	9.9 (95% CI 8.4-15)	48	<0.001	0.62	0.36-1.08	155	Variable duration	Sequential CTx (various regimens) and RTx (SBRT)
Mellon	2015	34.2	61	14	98	<0.001	-	-	159	Variable duration or NR	Sequential CTx (various regimens) and RTx (SBRT)
Reddy	2022	29.1	38	7	19	<0.001	0.3	0.12–0.91	57	NR	Sequential CTx (various regimens) and RTx (SBRT)
Teriaca	2021	24 (95% CI 21.6–26.3)	7	15 (95% CI 10–19.6)	32	0.03	-	-	50 (39 analysable)	Variable duration	Sequential CTx (FOLFIRINOX) and RTx (SBRT)
Zakem	2021	30	73	17	30	<0.0003	-	-	103	NR	Sequential CTx (various regimens) and RTx (SBRT)

Table S10: Overall survival of studies with heterogenous neoadjuvant regimens with or without resection

Abbreviations: CRTx: chemoradiotherapy, CTx: chemotherapy, NR: not reported, OS: overall survival, RTx: radiotherapy

Author	Year	OS – Resected (months)	n	OS – Unresected (months)	n	P-value	HR	CI	Total patients (n)	Treatment duration	Treatment
Bednar	2017	32 (22.3-not reached)	19	14.3 (11.2–17.5)	73	0.0002	0.261	0.123–0.551	92	NR	CTx, some sequential with RTx
Brada	2025	24 (95% CI 19-30)	103	-	-	-	-	-	103	Variable duration	CTx or concurrent CRTx
Fietkau	2020	R0: 26.5	25	16.5	87	0.003	-	-	180 (123 analysable)	Variable duration	CTx, some with sequential CRTx
		R1/R2/Rx: 16.9	11			NR					
Gemenetzis	2019	35.3 (95% CI, 24.5–46.0)	84	16.2 (95% CI, 15.2–17.3)	294	<0.001	-	-	415 (378 analysable)	NR	CTx, some with sequential RTx
Groot	2019	28 (25.5-30.5)	231	-	-	-	-	-	231	NR	CTx or concurrent CRTx
Hammel	2016	30.9 (95% CI, 12.3-not reached).	18	-	-	-	-	-	442 (18 analysable)	Variable duration	CT or concurrent CRTx with or without concurrent erlotinib
Herman	2015	22.2 (95%CI 13.6-not reached)	4	13.8 (95% CI 9.8-16.7)	45	0.182	0.45	0.13-1.49	49	Variable duration	RTx, some with sequential CTx
Hewitt	2022	29.9	41	12.2	117	NR	-	-	158	Variable duration	CTx, some with sequential CRTx
Hirono	2016	19.3	40	-	-	-	-	-	46 (40 analysable)	Variable duration	CTx or concurrent CRTx
Liang	2023	21.4	36	-	-	-	-	-	36	NR	CTx with or without sequential PD-1 inhibitor or RTx
Moningi	2015	20.2 (95 % CI 12.5–42.4)	19	12.3	69	0.07	-	-	88	NR	RTx, some with sequential CTx
Patel	2014	40.7 (12.9-124.9)	17	13.4 (5.9-63.6)	22	0.0002	-	-	52 (39 analysable)	NR	CTx or concurrent CRTx
Seelen	2022	24.7 (95% CI: 19.7–29.6)	168	-	-	-	-	-	168	NR	CTx or concurrent CRTx
Shrestha	2017	25.8	38	11.9	53	<0.0001	-	-	93 (91 analysable)	Variable duration	CTx, CRTx or CTx sequential with CRTx
Weisz Ejlsmark	2024	23.9 (95 % CI 21.2-not reached)	6	-	-	-	-	-	28 (6 analysable)	NR	RTx, some with sequential CTx
Wijetunga	2021	35 (95% CI 21.5-48.0)	60	-	-	-	-	-	60	Variable duration	CTx, some with sequential CRTx
Wolfe	2020	32.7 (95%CI 21.5-43.8)	72	-	-	-	-	-	72	Variable duration	CTx or concurrent CRTx, or CTx with sequential RTx

Table S11: Recurrence free survival or disease free survival following neoadjuvant chemotherapy with or without resection

Abbreviations: CTx: chemotherapy, DFS: Disease free survival, NR: not reported, RFS: Recurrence free survival

Author	Year	RFS/DFS – resected (months)	n	RFS/DFS – unresected (months)	n	P-value	HR	CI	Total (n)	Treatment duration	Treatment
Fossaert	2022	13.5	38	-	-	-	-	-	38	NR	CTx – various regimens
Lee	2024	22.7 (95% CI,17.9 to 27.5)	LAPC n = 98	-	-	-	-	-	662 (245 analysable)	NR	CTx – various regimens
		26.0 (95%CI 21.7 to 30.3)	BRPC n = 147								
Masui	2016	13.9	15	-	-	-	-	-	15	9 weeks	CTx - gemcitabine/S-1
Pietrasz	2019	13.5 (95% CI 9.7–17.3)	101	-	-	-	-	-	101	Variable duration	CTx - FOLFIRINOX
Su	2022	11.2 (95% CI, 7.2-20.2) (R0)	23/36	-	-	-	-	-	36 (34 analysable)	NR	CTx – various regimens
		3.8 (95% CI, 1.2-14.8) (R1)	11/36								
Yoo	2019	9.0 months (95% CI, 6.8–11.2)	135	-	-	-	-	-	135	NR	CTx – various regimens

Table S12: Recurrence free survival or disease free survival following neoadjuvant chemoradiotherapy with or without resection

Abbreviations: CRTx: chemoradiotherapy, DFS: Disease free survival, LR: limited pathologic response, nCR: near complete pathologic response, NR: not reported, pCR: complete pathologic response, RFS: Recurrence free survival

Author	Year	RFS/DFS – resected (months)	n	RFS/DFS – unresected (months)	n	P-value	HR	CI	Total (n)	Treatment duration	Treatment
He	2018	pCR: 26	18	-	-	-	-	-	182	NR	Concurrent CRTx – various regimens
		nCR: 12	29								
		LR: 12	135								
Okamura	2024	11	26	-	-	-	-	-	26	6 weeks	Concurrent CRTx with S-1

Table S13: Recurrence free survival or disease free survival following neoadjuvant sequential chemotherapy and chemoradiotherapy with or without resection

Abbreviations: CRTx: chemoradiotherapy, CTx: chemotherapy, DFS: Disease free survival, RFS: Recurrence free survival

Author	Year	RFS/DFS – resected (months)	n	RFS – unresected (months)	n	P-value	HR	CI	Total (n)	Treatment duration	Treatment
Truty	2021	23.5	194	-	-	-	-	-	194	Variable duration	Sequential CTx and CRTx – various regimens
Pietrasz	2019	17.7 (95% CI 9.9–25.5)	102	-	-	-	-	-	102	Variable duration	Sequential CTx (FOLFIRINOX) and CRTx (various regimens)
Yabushita	2023	17.5	94	-	-	-	-	-	122 (94 analysable)	~6 weeks	Sequential CTx (gemcitabine/S-1) and CRTx with S-1

Table S14: Recurrence free survival or disease free survival from studies with heterogenous neoadjuvant regimens with or without resection

Abbreviations: CRTx: chemoradiotherapy, CTx: chemotherapy, DFS: Disease free survival, NR: not reported, RFS: Recurrence free survival, RTx: radiotherapy

<table>
<tr><th>Author</th><th>Year</th><th>RFS/DFS-resected (months)</th><th>n</th><th>RFS/DFS – unresected (months)</th><th>n</th><th>P-value</th><th>HR</th><th>CI</th><th>Total (n)</th><th>Treatment duration</th><th>Treatment</th></tr>
<tr><td>Brada</td><td>2025</td><td>13 (95% CI 9-17)</td><td>103</td><td>-</td><td>-</td><td>-</td><td>-</td><td>-</td><td>103</td><td>Variable duration</td><td>CTx or concurrent CRTx</td></tr>
<tr><td rowspan="2">Fietkau</td><td rowspan="2">2021</td><td>R0: 16.6</td><td>25</td><td rowspan="2">11.9</td><td rowspan="2">87</td><td>0.003</td><td rowspan="2">-</td><td rowspan="2">-</td><td rowspan="2">180 (123 analysable)</td><td rowspan="2">Variable duration</td><td rowspan="2">CTx or concurrent CRTx</td></tr>
<tr><td>R1/R2/Rx: 11</td><td>11</td><td>NR</td></tr>
<tr><td>Gemenetzis</td><td>2019</td><td>11.3 (95% CI 9.3–13.3)</td><td>84</td><td>-</td><td>-</td><td>-</td><td>-</td><td>-</td><td>84</td><td>NR</td><td>CTx some with sequential RTx</td></tr>
<tr><td>Groot</td><td>2019</td><td>9.8 (8.3-11.3)</td><td>231</td><td>-</td><td>-</td><td>-</td><td>-</td><td>-</td><td>231</td><td>NR</td><td>CTx or concurrent CRTx</td></tr>
<tr><td>Liang</td><td>2023</td><td>13.6</td><td>36</td><td>-</td><td>-</td><td>-</td><td>-</td><td>-</td><td>36</td><td>NR</td><td>CTx with or without sequential PD-1 inhibitor or RTx</td></tr>
</table>

Peng	2019	22.3 (complete/marked response)	16/71	-	-	-	-	-	71	NR	Concurrent CRTx, CTx, or CTx with sequential CRTx
		14.3 (moderate response)	42/71								
		15.2 (minimal response)	12/71								
Seelen	2023	11.0 (8.2–13.8)	168	-	-	-	-	-	168	NR	CTx or concurrent CRTx
Shrestha	2017	13	38	-	-	-	-	-	93 (38 analysable)	Variable duration	CTx, CRTx or CTx sequential with CRTx
Wijetunga	2021	30 (95% CI 16.5-44.1)	60	-	-	-	-	-	60	Variable duration	CTx, some with sequential CRTx

Table S15: Progression free survival following neoadjuvant chemotherapy with or without resection

Abbreviations: CTx: chemotherapy, NR: not reported, PFS: progression free survival

Author	Year	PFS – Resected (months)	n	PFS - Unresected (months)	n	P-value	HR	CI	Total patients (n)	Treatment duration	Treatment
He	2021	10.6	45	5.7 (95% CI, 3.8-7.7)	31	0.009	0.562	0.303–1.044	140 (76 analysable)	NR	CTx – various regimens
Li	2019	19.3	12	11.9	27	NR	-	-	41 (39 analysable)	At least 8 weeks	CTx - mFOLFIRINOX
Su	2022	14.2 (95% CI, 12.6-19.4)	34	6.6 (95% CI, 5.2-8.3)	83	0.00041	-	-	117	NR	CTx – various regimens
Templeton	2021	17	5	9 (95% CI, 7.5–24.5)	15	0.016	-	-	20	Median 12 weeks	CTx – various regimens
Yoo	2019	13.4 (95% CI, 12.5–14.4)	135	-	-	-	-	-	135	NR	CTx – various regimens

Table S16: Progression free survival following neoadjuvant concurrent chemoradiotherapy with or without resection

Abbreviations: CRTx: chemoradiotherapy, NR: not reported, PFS: progression free survival

Author	Year	PFS – Resected (months)	n	PFS - Unresected (months)	n	P-value	HR	CI	Total patients (n)	Treatment duration	Treatment
Chakraborty	2014	13 (95% CI 4.4–not reached).	5	2.1 months (95% CI 1.5–2.4).	8	NR	-	-	13	4 weeks	Concurrent CRTx with capecitabine
Okamura	2024	12.5	26	-	-	-	-	-	26	6 weeks	Concurrent CRTx with S-1
Takahashi	2022	21.6 (95% CI 10.9–40.9)	29	-	-	-	-	-	52 (29 analysable)	5.5 weeks	Concurrent CRTx with S-1

Table S17: Progression free survival following neoadjuvant sequential chemotherapy and radiotherapy with or without resection

Abbreviations: CTx: chemotherapy, NR: not reported, PFS: progression free survival, RTx: radiotherapy, SBRT: stereotactic body radiotherapy

Author	Year	PFS - Resected (months)	n	PFS - Unresected (months)	n	P-value	HR	CI	Total patients (n)	Treatment duration	Treatment
Hill	2022	27.7 (local PFS)	107	-	-	-	-	-	107	Variable duration	Sequential CTx (various regimens) and RTx (SBRT)
Reddy	2022	12.9	38	1.6	19	<0.001	0.4	0.17–0.93	57	NR	Sequential CTx (various regimens) and RTx (SBRT)
Mellon	2015	23.1	61	9.5	98	<0.001	-	-	159	Variable duration or NR	Sequential CTx (various regimens) and RTx (SBRT)

Table S18: Progression free survival of studies with heterogenous neoadjuvant regimens with or without resection

Abbreviations: CRTx: chemoradiotherapy, CTx: chemotherapy, PFS: progression free survival, NR: not reported, RTx: radiotherapy

Author	Year	PFS - Resected (months)	n	PFS - Unresected (months)	n	P-value	HR	CI	Total patients (n)	Treatment duration	Treatment
Bednar	2017	15	19	-	-	-	-	-	92 (19 analysable)	NR	CTx, some with sequential RTx
Blazer	2015	18 (CI 95% 11.9 – not reached)	22	8 (CI 95% 4.5 - 10.4)	21	<0.001	-	-	43	Variable duration	CTx or concurrent CRTx
Hirono	2016	13.2	40	-	-	-	-	-	46 (40 analysable)	Variable duration	CTx or concurrent CRTx
Wolfe	2020	17.8 (95%CI 14.4-21.1)	72	-	-	-	-	-	72	Variable duration	CTx or concurrent CRTx, or CTx with sequential RTx

Table S19: Quality assessment of single-arm studies using Methodological Index for Non-Randomised Studies (MINORS) tool

	A clearly stated aim	Inclusion of consecutive patients	Prospective collection of data	Endpoints appropriate to the aim of the study	Unbiased assessment of the study endpoint	Follow-up period appropriate to the aim of the study	Loss to follow up less than 5%	Prospective calculation of the study size	Overall
Amodeo et al. (2018) [44]	2	1	2	2	1	2	2	0	12
Belfiore et al. (2015) [46]	2	1	2	2	1	1	2	0	11
Chakraborty et al. (2014) [52]	2	1	2	2	1	2	1	0	11
Goji et al. (2015) [63]	2	1	2	2	1	2	2	1	13
Hill et al. (2022) [43]	2	1	0	2	1	2	2	0	10
Leen et al. (2018) [78]	2	2	0	2	1	2	2	0	11
Li et al. (2019) [79]	2	1	2	2	1	2	1	0	11
Murphy et al. (2019) [88]	2	1	2	2	1	2	2	0	12
Nagakawa et al. (2017) [89]	2	1	2	2	1	2	2	0	12
Okamura et al. (2024) [92]	2	2	0	2	1	2	2	0	11
Paik et al. (2015) [93]	2	0	0	2	1	2	2	0	9
Philip et al. (2020) [97]	2	1	2	2	1	2	1	2	13
Reddy et al. (2022) [99]	2	0	0	2	1	2	2	0	9
Saito et al. (2017) [101]	2	0	0	2	1	1	2	0	8
Stein et al. (2016) [105]	2	1	2	2	1	2	2	0	12
Sui et al. (2017) [108]	2	0	0	2	1	2	2	0	9
Takahashi et al. (2016) [109]	2	1	2	2	1	2	2	0	12
Takahashi et al. (2022) [110]	2	1	2	2	1	2	2	2	14
Tasu et al. (2024) [111]	2	1	2	2	1	2	2	2	14
Temraz et al. (2022) [113]	2	1	2	2	1	2	2	2	14
Teriaca et al. (2021) [114]	2	1	2	2	1	2	2	0	12
Tsujimoto et al. (2019)	2	0	0	2	1	2	2	0	9
Yabushita et al. (2023) [120]	2	1	2	2	1	2	2	2	14
Yamaguchi et al. (2022) [121]	2	1	2	2	1	2	2	2	14

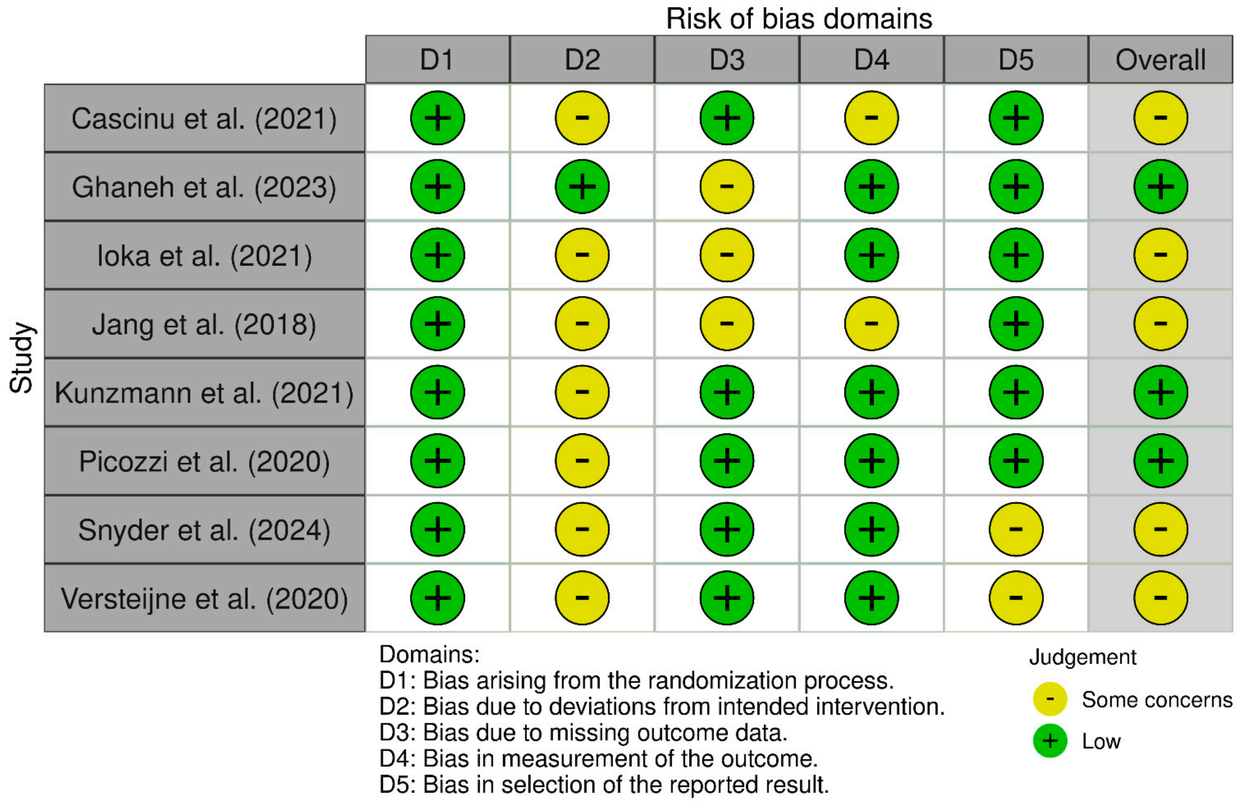

Figure S1: Risk of bias assessment of included articles using the Version 2 of the Cochrane Risk-of-Bias Tool for Randomised Trials (RoB 2) tool [32, 51, 62, 72, 73, 75, 104, 117].

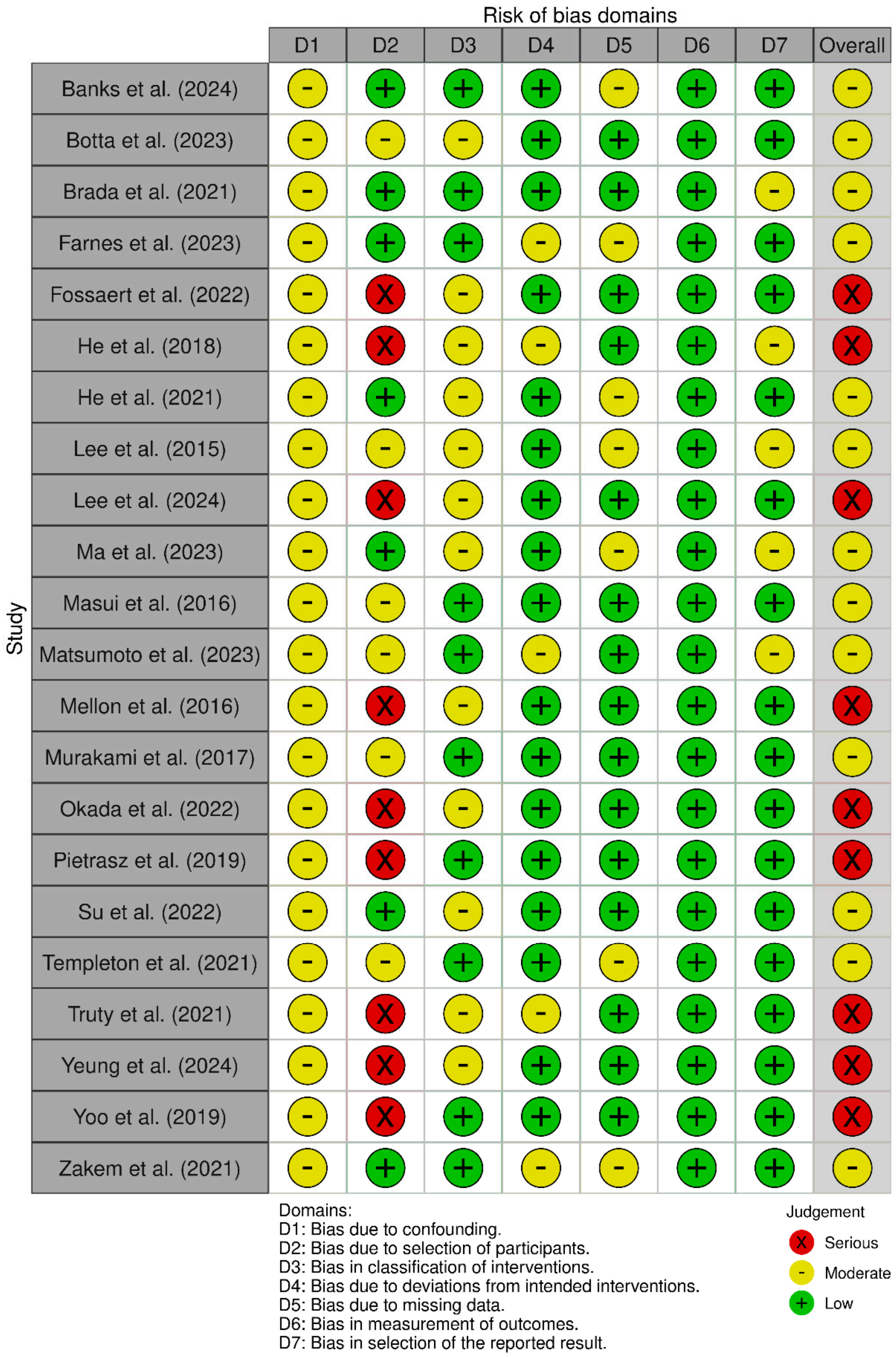

Figure S2: Risk of bias assessment of included articles using the Risk of Bias in Non-Randomised Studies of Interventions (ROBINS-I) tool [28, 42, 48, 49, 61, 67, 68, 76, 77, 81-83, 85 87, 91, 98, 106, 112, 115, 122-124].

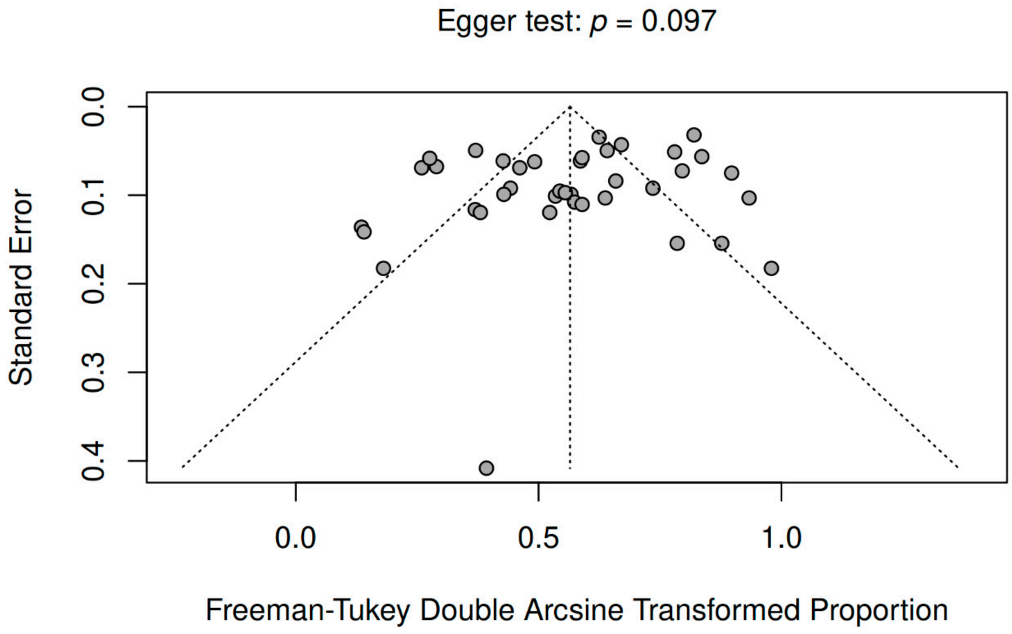

Figure S3: Assessment of small study effects with funnel plot and Egger's test for objective response rate (n=29). Plots representing individual studies.

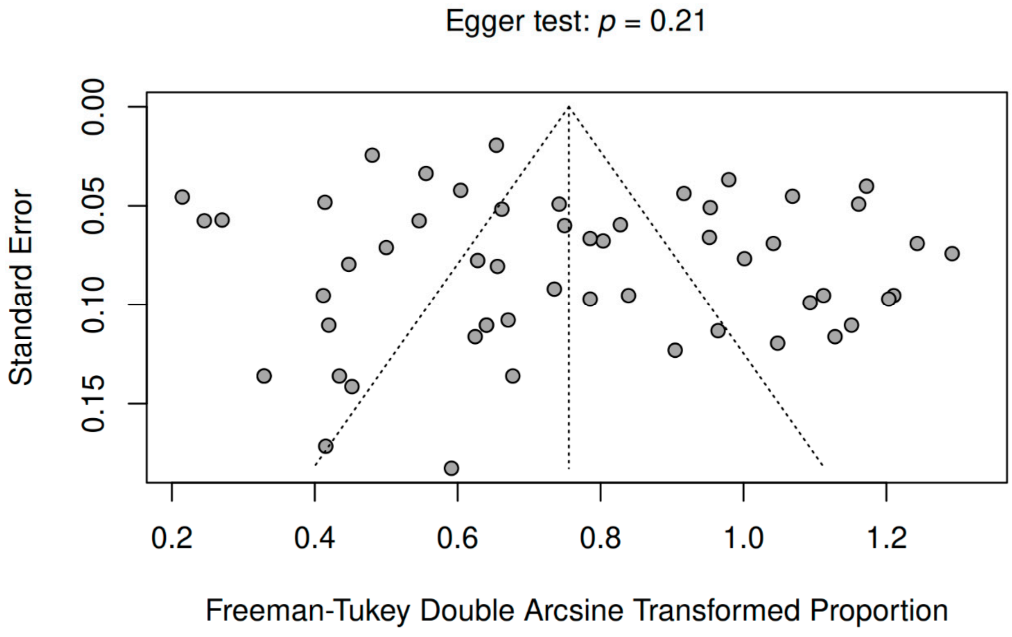

Figure S4: Assessment of small study effects with funnel plot and Egger's test for rates of proceeding to surgery (n=43). Plots representing individual studies.

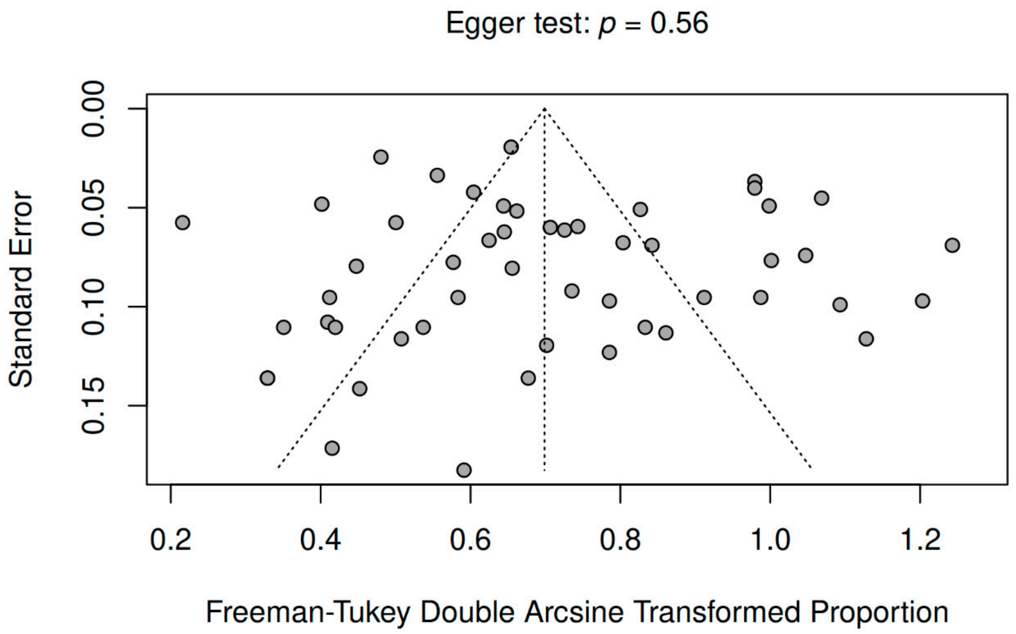

Figure S5: Assessment of small study effects with funnel plot and Egger's test for rates of resection (n=39). Plots representing individual studies.

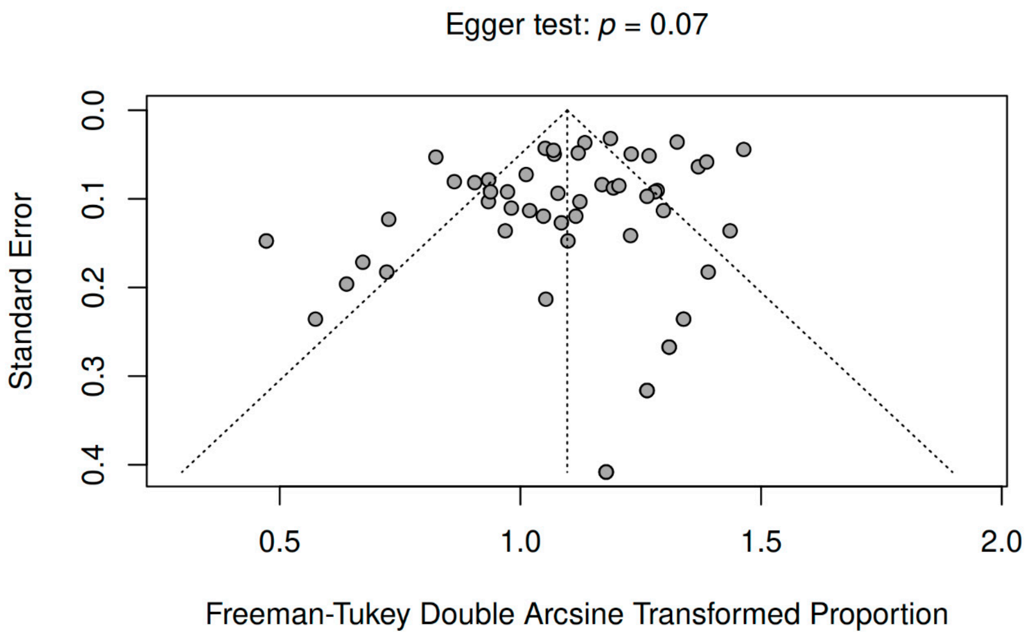

Figure S6: Assessment of small study effects with funnel plot and Egger's test for resection status (n=45). Plots representing individual studies.

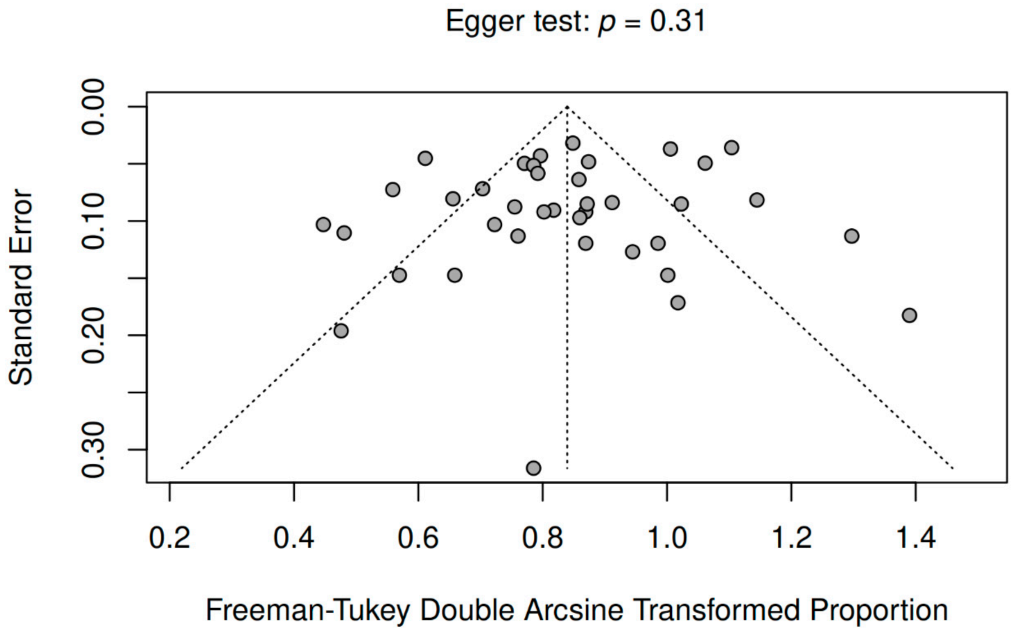

Figure S7: Assessment of small study effects with funnel plot and Egger's test for nodal status (n=30). Plots representing individual studies.
